# A near complete genome for goat genetic and genomic research

**DOI:** 10.1186/s12711-021-00668-5

**Published:** 2021-09-10

**Authors:** Ran Li, Peng Yang, Xuelei Dai, Hojjat Asadollahpour Nanaei, Wenwen Fang, Zhirui Yang, Yudong Cai, Zhuqing Zheng, Xihong Wang, Yu Jiang

**Affiliations:** Key Laboratory of Animal Genetics, Breeding and Reproduction of Shaanxi Province, College of Animal Science and Technology, Northwest A&F University, Xinong Rd 22, Yangling, 712100 Shaanxi China

## Abstract

**Background:**

Goat, one of the first domesticated livestock, is a worldwide important species both culturally and economically. The current goat reference genome, known as ARS1, is reported as the first nonhuman genome assembly using 69× PacBio sequencing. However, ARS1 suffers from incomplete X chromosome and highly fragmented Y chromosome scaffolds.

**Results:**

Here, we present a very high-quality de novo genome assembly, Saanen_v1, from a male Saanen dairy goat, with the first goat Y chromosome scaffold based on 117× PacBio long-read sequencing and 118× Hi-C data. Saanen_v1 displays a high level of completeness thanks to the presence of centromeric and telomeric repeats at the proximal and distal ends of two-thirds of the autosomes, and a much reduced number of gaps (169 vs. 773). The completeness and accuracy of the Saanen_v1 genome assembly are also evidenced by more assembled sequences on the chromosomes (2.63 Gb for Saanen_v1 vs. 2.58 Gb for ARS1), a slightly increased mapping ratio for transcriptomic data, and more genes anchored to chromosomes. The eight putative large assembly errors (1 to ~ 7 Mb each) found in ARS1 were amended, and for the first time, the substitution rate of this ruminant Y chromosome was estimated. Furthermore, sequence improvement in Saanen_v1, compared with ARS1, enables us to assign the likely correct positions for 4.4% of the single nucleotide polymorphism (SNP) probes in the widely used GoatSNP50 chip.

**Conclusions:**

The updated goat genome assembly including both sex chromosomes (X and Y) and the autosomes with high-resolution quality will serve as a valuable resource for goat genetic research and applications.

**Supplementary Information:**

The online version contains supplementary material available at 10.1186/s12711-021-00668-5.

## Background

As one of the first domesticated livestock, goat is considered the ‘poor man's cow’ due to its significant contribution to the livelihood of rural communities in many developing and underdeveloped countries. The global number of goats exceeds 1 billion and continues to increase (FAO 2019). The first goat reference genome (CHIR_1.0) was generated from a female Yunnan black goat in 2013 [1], followed by minor (CHIR_1.1) and major (CHIR_2.0) changes in 2014 [2] and 2015, respectively. CHIR_1.0 represents the first application of the optical mapping technology to genome assembly scaffolding and has served as a valuable resource for gene mapping and marker-assisted breeding in goats. This assembly further enabled the design of the first 50K GoatSNP50 chip [3] which has been extensively used to study genetic diversity in domestic goats [4–9] and its effect on phenotypic variation [10–12].

The update of the goat reference genome assembly in 2016, named ARS1 [13, 14], was the first nonhuman genome assembly generated by the PacBio sequencing technology and provided a roadmap to produce reference-quality genome sequences with affordable cost and improved scalability. Nevertheless, ARS1 was generated with a 69× coverage of PacBio sequencing data, suggesting that the X and Y chromosomes were each represented by a ~ 35 × coverage only. Likely due to this lower depth of haploid coverage, the ARS1 reference genome lacks solitary scaffolds for the X and Y chromosomes. Sex chromosomes are critical to sexual development and fertility [15, 16]; furthermore, the Y chromosome is especially useful in inferring population genetics. Therefore, it would be highly beneficial to generate complete assemblies for the goat X and Y chromosomes. In addition, our previous analysis reported at least 38.3 Mb of non-reference sequences in ARS1 due to either structural variations or assembly errors [17]. Therefore, ARS1 could be greatly improved to enhance the discovery of genetic variants, design of SNP chips, genome-wide association studies (GWAS) and implementation of genomic selection.

The long-read sequencing technologies continue to evolve [18–20], delivering highly accurate long reads and increased capability with reduced costs. To generate a high-quality reference genome for goats, particularly with continuous assemblies for the sex chromosomes, we sequenced a Saanen buck using PacBio long-read sequencing with high depth in combination with Hi-C technologies for scaffolding. Our new genome assembly displays excellent continuity and completeness, and will serve as a valuable reference assembly for future research in goat genetic and genomic studies.

## Methods

### Genome sequencing

A two-year-old Saanen buck from a dairy farm in Shaanxi province of China was chosen for sequencing. The animal was healthy, and no genetic defects were recorded for it and its parents. A QIAamp DNA Mini Kit (Qiagen, Hilden, Germany) was used to purify DNA according to the manufacturers’ instructions. High-quality genomic DNA was extracted from the liver tissue and subjected to sequencing using the PacBio sequel II and Illumina HiSeq X Ten platforms at the genome center of Frasergen Bioinformatics Co., Ltd (Wuhan, China). PacBio sequencing was conducted using the Sequel Binding Kit 1.0, Sequel Sequencing Kit 1.0 and Sequel SMRT Cell 8M (Pacific Bioscience, Menlo Park, USA) on the PacBio sequel II platform. Three SMRT Cells were employed for genome sequencing and SMRT LINK 5.0 was used to filter the raw data from the zero-mode waveguide.

An Illumina library with insert sizes of 400 bp was constructed using an Illumina TruSeq Nano DNA Library Prep Kit (Illumina, San Diego, USA) and then sequenced on an Illumina HiSeq X Ten instrument in paired-end mode with read lengths of 150 bp. The raw sequencing data were filtered by fastp v0.12.3 [21] to generate clean reads, using default parameters except for a quantified Phred quality score of at least 20 (−q 20) and a minimum length of 100 bp for clean reads (−l 100).

### De novo genome assembly

MECAT2 v20190314 [22] was used for error correction of the raw PacBio long reads. For de novo genome assembly, we initially created two contig assemblies: one using Flye v2.8 [23] with default parameters except that corrected reads were used as input (–pacbio-corr), and a second using Wtdbg2 v2.5 [24]. The wgdbg2 module was used to assemble raw reads and generate the contig layout and edge sequences, followed by the wtpoa-cns module to produce the final consensus contigs. Our results suggest that the two versions demonstrated similar continuity (contig N50 33.9 Mb for Flye vs. 35.3 Mb for Wtdbg2). After sequence polishing by PacBio long reads and Illumina short-reads as described below, the resulting Flye assembly displayed better BUSCO completeness than the Wtdbg2 version (94.0% vs. 93.1%).

Considering that the Y chromosome is the most difficult part of the genome to sequence and assemble, to obtain more continuous assemblies of the sex chromosomes, we specifically examined the continuity of the Y chromosome-linked scaffolds of the two versions. The single-copy region of the Y chromosome is conserved, especially among related species [25]. Thus, we used the BLAST-Like Alignment Tool (BLAT) [26] to extract scaffolds containing the ten Y chromosome-linked single-copy genes (*AMELY*, *OFD1Y*, *USP9Y*, *ZRSR2Y*, *UTY*, *DDX3Y*, *ZFY*, *EIF2S3Y*, *SRY*, and *RBMY*) in sheep [27] and found that one scaffold from the Flye version contains seven of these loci in the same order as on the ovine Y chromosome. We also show that the longest scaffold from Wtdbg2 only covers five of the mentioned single-copy genes, indicating that the Y chromosome-linked genes from Flye would be more continuous. Therefore, the Flye contig version was used as the basis for all subsequent refinements.

The raw PacBio reads were mapped to the contigs using minimap2 v2.17 [28] with the settings recommended for PacBio sequencing data (-cx map-pb–secondary = no). Racon was then used to polish the contigs with two iterations [29]. In addition, the paired-end Illumina sequencing reads (81× , PE150 bp) were used to polish the assembly using the Pilon v1.20 tool [30]. Iterative polishing by Pilon was achieved by aligning Illumina reads to the corresponding assembly or polishing consensus sequence from the previous iteration using the BWA MEM v0.7.13-r1126 alignment algorithm [31]. The resulting alignment file was sorted by Samtools v1.3 [32] and then subjected to Pilon together with the corresponding assembly to generate the consensus sequence. Pilon was run with default settings to fix bases and small indels (–fix snps, indels). The resulting polished de novo assembly was 2.69 Gb long with the contig N50 being 34.0 Mb long.

To obtain a chromosome-level genome assembly, one Hi-C library was constructed for sequencing (see Additional file 1: Supplementary methods) for the detailed Hi-C library preparation protocol. DNA from blood of the same individual used for genome assembly was extracted for Hi-C library construction. A restriction enzyme (MboI) was used to digest the cross-linked DNA. The cross-linked DNA was unlinked using a protease, and the chimeric junctions of the genome were sheared to a size of 300–500 bp. An Illumina library with an approximately 300-bp insert size was constructed according to the Illumina library preparation protocol (Illumina Inc., San Diego, CA, USA). Sequencing of the Hi-C library was also performed using the Illumina HiSeq X Ten instrument in paired-end mode with read lengths of 150 bp.

The Hi-C paired-end reads were aligned to the contigs using the Juicer software v1.5 [33] to obtain the interaction matrix. Subsequently, 3D-dna v180419 [34] was applied to order and orient the contigs. Finally, Juicebox 1.11.08 [35] was used to manually adjust the position of the contigs based on Hi-C heatmaps. PBjelly v1.01 [36] with default parameters was used to close the gaps in the resulting scaffolds using PacBio long reads, yielding the final version of the Saanen goat assembly (Saanen_v1).

### Estimation of the genome size

The genome size of Saanen_v1 was estimated using the gce-1.0.2 script [37], which is a kmer-based approach. In total, 97 Gb Illumina clean paired-end reads from Saanen_v1 were used. The 17-kmer distribution showed a major peak at 71 × (see Additional file 2: Figure S1). Based on the number of kmers and relative kmer depth, we estimated the genome size of goats to be 2.72 Gb, according to the formula: Genome size = kmer_number/Peak_depth [37].

### Detection of structural variations

Whole-genome alignment between the Saanen_v1 and ARS1 genome assemblies was performed using the nucmer script from MUMmer v3.23 [38] with the following options: –maxmatch–l 100–c 500. The output of nucmer was then analyzed using Assemblytics [39] to detect structural variations (between 50 bp and 10,000 bp) and only variations larger than 10 bp in either the ref_gap_size or the query_gap_size field were retained for further analysis. Functional annotation of the structural variants was conducted using ANNOVAR [40].

### Gene annotation

A combination of ab initio gene prediction, homology-based prediction and RNA-seq assisted gene prediction was used to comprehensively annotate genes in Saanen_v1. For ab initio gene prediction, we used Augustus with the model trained by BRAKER v2.1.5 [41] with extrinsic transcriptome evidence provided as hints; SNAP v2006-07–28 [42] was also used to generate ab initio gene prediction. For homology-based prediction, protein sequences from different species including cattle (*Bos taurus*), goat (*Capra hircus*), sheep (*Ovis aries*) and pig (*Sus scrofa*) were selected and processed with Exonerate v.2.2.0 [43] to find the best result per protein sequence. For RNA-seq assisted gene prediction, data from different sources, including publicly available transcriptomic data from the NCBI SRA database, our unpublished Illumina short read RNA-seq and PacBio long-read RNA sequences (Iso-Seq) from the testicular tissue of the individual used to generate Saanen_v1, and additional PacBio Iso-Seq data (abomasum tissue) from three Shanbei white Cashmere goats were used. All RNA-seq data were then mapped to the Saanen_v1 assembly with STAR v.2.7.3a [44], followed by StringTie v2.0 [45] and TransDecoder v5.5.0 (https://github.com/TransDecoder), which were combined to find the coding regions. IsoSeq v.3.2.2 was used to process Iso-Seq data (https://github.com/PacificBiosciences/IsoSeq), and then TAMA [46] was applied for transcriptomic annotation using Iso-seq data. Finally, EvidenceModeler v1.1.1 [47] was used to integrate all evidence into a non-redundant gene annotation.

### Annotation of repeats

Interspersed repeats and low complexity DNA sequences were identified using RepeatMasker v4.0.7 (http://www.repeatmasker.org) with a combined repeat database including Dfam v.20170127 and RepBase v20170127 with parameters: -species Ruminantia-xsmall-s-no_is -cutoff 255 -frag 20000 -gff.

To identify telomeric repeats, we used a strategy similar to that reported previously [13]. First, we searched for the 6-mer vertebrate motif (TTAGGG) and looked for all exact matches in the assembly. We also ran DUST [48] with a window size of 64 bp and threshold of 20 bp to identify low-complexity regions. Windows with at least 10 consecutive identical 6-mer matches (forward or reverse strand) were merged with the intersecting low-complexity regions. Those regions that were at least 2 kb long with a hexamer density higher than 0.5, were then retained as potential telomeres. To identify putative centromeric features in the assembly, we considered as putative centromeric sequences, those in which the repeat class/family was flagged as “Satellite/centr” by Repeatmasker and longer than 5 kb.

### Assessment of the quality of the assembly

The completeness and accuracy of the assembly were assessed using BUSCO v3.0.2 [49] in protein mode with the lineage dataset mammalia_odb9 containing 4,104 single-copy orthologues.

The GC content was calculated in 1-kb windows using the “nuc” subcommand from bedtools v2.25.0 [50]. Sequencing depth was calculated based on the alignment BAM file in 1-kb windows using the “depth” subcommand from Sambamba v0.6.7 [51].

For the comparison of assembly continuity, we downloaded reference genome assemblies including human (GRCh38, GCA_000001405.28), pig (Sscrofa11.1, GCA_000003025.6), goat (ARS1, GCA_001704415.1), cattle (ARS-UCD1.2, GCA_002263795.2), buffalo (UOA_WB_1, GCA_003121395.1), sheep (Oar_rambouillet_v1.0, GCA_002742125.1) and horse (EquCab3.0, GCA_002863925.1) from NCBI GenBank. The previous goat reference genome (CHIR_2.0, GCA_000317765.2) was also downloaded.

We compared the mappability of sequence reads between the Saanen_v1 and ARS1 genomes from whole-genome sequencing, RNA-seq and Iso-seq data. Illumina short-read data were mapped to the reference genome using BWA-MEM v.0.7.13-r1126 [31]. RNA-seq data were mapped to the reference genome using HISAT2 [52]. Iso-seq data were first processed using the IsoSeq v3 workflow (https://github.com/PacificBiosciences/IsoSeq) to obtain the full length reads (FLNC reads) and then aligned to the reference genome using GMAP [53], to count the number of mapped reads (identity > 0.99 and coverage > 0.95).

Approximately 40× whole-genome sequencing data from one sample of Yunnan black goat were aligned to the Saanen_v1 and ARS1 genome assemblies. Then, FRCBam [54] was used to evaluate compression/expansion (CE) errors.

The quality value (QV) of each assembly was estimated following a previous protocol [55]. In brief, FreeBayes [56] was used to determine the polymorphic sites and QV was calculated using the formula QV = −10*log_10_(substitution_number/genome_size) (https://github.com/lloydlow/BuffaloAssemblyScripts/tree/master/QV_estimation). In addition to their own whole-genome sequencing data, we downloaded another three datasets from one Asian (ERR313211), one European (SRR5803234) and one African (SRR5803191) individual for QV estimation.

### Annotation of long non-coding RNA (lncRNA) and miRNA

For lncRNA prediction, RNA-seq alignments were first used to assemble transcripts from each dataset, which were then merged into a unique set of transcripts using Cuffmerge (Cufflinks v2.1.1) [57]. Transcripts longer than 200 nucleotides were removed and the remaining transcripts were compared with the NCBI goat gene annotation to remove transcripts that overlapped with known protein coding and noncoding genes (mRNA, tRNA, rRNA, snRNA, snoRNA, miRNA) using Cuffcompare (Cufflinks v2.1.1). PLEK v1.2 [58] and CPC2 v0.1 [59] were used with default parameters to determine the candidate non-coding transcripts. Those candidate lncRNAs were then blasted against the NCBI nr database to remove hits with more than 90% identity and more than 50% coverage.

For microRNA annotation, nine miRNA-seq datasets (see Additional file 3: Table S1) were downloaded from the NCBI SRA database. miRDeep2 v2.0.0.5 [60] was used to identify known goat miRNAs from the miRBase v22 database [61] and to predict novel miRNAs. A miRDeep2 score cut-off of 5 was used as recommended by the authors of the software [60], corresponding to a true positive prediction percentage greater than 95%, and a signal-to-noise ratio higher than 20.

### Gene annotation mapping

We chose the recently developed mapping tool Liftoff [62] for gene annotation mapping, which was shown to be the only tool that could map nearly all the human genes from one individual to another [63]. Liftoff takes all the genes and transcripts from a genome assembly and maps them, chromosome by chromosome, to another assembly. In the case of genes that fail to map to the same chromosome, Liftoff attempts to map them across chromosomes. It does not rely on whole-genome alignment, but instead, it maps each gene individually at the transcript level with high accuracy and efficiency. By using this mapping tool, we reciprocally mapped the gene annotations between the Saanen_v1 and ARS1 assemblies. A gene annotation was considered as successfully mapped to another assembly if the mapping identity was more than 95% and the coverage greater than 90%.

### Discovery of large assembly errors

We further used Minimap2 v.2.17-r941 [28] to perform whole-genome alignment and check whether there were disagreements between the Saanen_v1 and ARS1 genome assemblies. To verify whether those disagreements could represent true structural variations or assembly errors in either of the assemblies, we aligned Saanen_v1 and ARS1 to two additional assemblies (CHIR_2.0 and Oar_rambouillet_v1.0) to explore which of them could be supported by these two additional assemblies.

Furthermore, we downloaded the Hi-C data of ARS1 to generate its Hi-C contact matrix. Most of the large assembly errors (> 1 Mb) from ARS1 could be visually confirmed by disagreement in the Hi-C contact heatmap whereas the correct assembly region in Saanen_v1 could also be supported by its own Hi-C contact heatmap.

The alignment file in PAF format generated by Minimap2 [28] was visualized using Ribbon [64] for collinearity or D-genies [65] for dotplots.

### Identification of discrepancies in SNP positions

Probe sequences of the GoatSNP50 chip were derived from the marker manifest files provided by the International Goat Genome Consortium (http://www.goatgenome.org). The probes were mapped to the Saanen_v1 and ARS1 assemblies using BLAST v2.2.31 [66] and those with an identity level greater than 95% and a coverage greater than 90% were considered as mapped. All mapping coordinates were obtained from the output of BLAST searches.

The SNP positional discrepancies were classified into three categories: (1) uniquely mapped to Saanen_v1; (2) assigned to one chromosome in Saanen_v1, but to another chromosome/scaffold in ARS1; and (3) assigned to the same chromosome in both Saanen_v1 and ARS1 assemblies, but with a changed order of the index. For those in group 3, we first indexed the probes by their positions on the Saanen_v1 assembly and by an in-house Python script to examine whether the order of their index had changed when mapped to ARS1. Those with a changed order were then reported as one type of SNP positional discrepancy. Since we mainly focused on SNP positional improvement in the Saanen_v1 assembly, we found 13 probes that were uniquely mapped to ARS1, which were most likely due to the presence of ARS1-specific sequences that were not included in the list of SNP positional discrepancies.

### Analysis of substitution rate

The multiple sequence alignment of the autosomes for cattle, yak, sheep and goat was generated with LAST (-m100-E0.05) [67] and MULTIZ [68] by using cattle as the reference genome, which was provided by our Ruminant Genome Project (http://animal.nwsuaf.edu.cn/code/index.php/RGD). For the Y chromosome, we generated the multiple sequence alignment using the same pipeline. The Y chromosome sequences for sheep (CM022046.1), cattle (CM001061.2) and yak (CM016720.1) were downloaded from NCBI. The multiple sequence alignments in maf format were subjected to the following manipulations before estimation of the substitution rate. To obtain conservative estimates of the substitution rate, duplicates were removed from the alignment using MAFDUPLICATEFILTER from the MAFTOOLS suite [69]. All alignment blocks were then converted to the positive strand of the ancestral sequence (maf_flip_for_ref.py available at https://github.com/makovalab-psu/great-ape-Y-evolution). Again, to obtain conservative estimates, we only kept alignment blocks in which all four species were present, thus largely restricting our analysis to X-degenerate regions (645.1 kb) for the Y chromosome and 1.96 Gb for autosomes.

The final filtered alignment was then used to pick the best-fitted substitution model using JMODELTEST [70]. The GTR (also called REV) model with variable substitution rates (–nrates = 4) was chosen. Using this model and our filtered alignment, we ran PHYLOFIT [71] with the following settings: phyloFit-E-subst-mod REV-nrates 4-tree " ((cattle,yak), (sheep,goat))" to estimate the substitution rates for the autosomes, X and Y chromosomes, respectively. Finally, the male-to-female mutation rate ratio (α_m_) was estimated on the Y-to-autosomal substitution rate using the equation Y/A = α_m_/(1 + α_m_).

## Results

### De novo assembly of the Saanen dairy goat genome

We chose a Saanen buck and extracted high-molecular-weight DNA from liver tissue. Then, we performed single molecular real-time (SMRT) long-read sequencing using PacBio RSII at 117× coverage (327.7 Gb) and obtained 17.1 M subreads from three libraries with subread N50 length ranging from 29.8 to 31.2 kb (see Additional file 3: Table S2). In addition, we sequenced the same individual using an Illumina HiSeq X Ten and generated 228.8 Gb short reads (81× coverage).

The raw PacBio long reads were first subjected to read correction using MECAT2 [22] followed by de novo assembly using Flye [23] which can generate highly continuous and complete assemblies by constructing accurate repeat graphs. To improve base accuracy, the assembly was corrected by one round of Racon [29] using the PacBio long reads [29] and then two rounds of Pilon [30] using the Illumina whole-genome short-read data [30] (see Methods). The resulting genome assembly included 1684 contigs summing up to 2.69 Gb (see Additional file 3: Table S3).

**Fig. 1:**
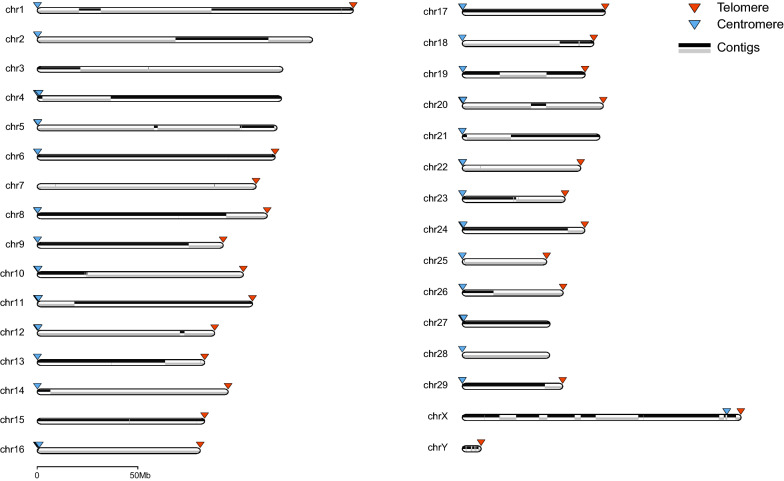
Chromosomal distribution of contigs of the Saanen_v1 genome assembly. Triangles represent putative telomere signals (red) and centromere sequences (blue)

We further generated 118 × Hi-C data (330.8 Gb) from the same individual to scaffold the contigs. The gaps in the scaffolds were closed by PBjelly [36] using the PacBio long reads, which successfully filled 223 gaps, extending 85 gaps at both ends and 982 gaps at only one end. The final scaffolded assembly (hereafter referred to as Saanen_v1) had a total length of 2.69 Gb with a scaffold N50 length of 102.3 Mb, which is greater than that of the present goat reference genome, ARS1 (87.2 Mb). The final contig N50 length was 46.2 Mb which is also much longer than that of ARS1 (26.2 Mb), and most reference assemblies of livestock species (see Additional file 2: Figure S2). Notably, Saanen_v1 possessed far fewer gaps than ARS1 (Saanen_v1: 169 vs. ARS1: 773). For Saanen_v1, 27 of the 31 chromosomes had fewer than 10 gaps including three gapless chromosomes, i.e., chr17, 27 and 28 (Table 1 and Fig. 1), which shows its high continuity. Furthermore, Saanen_v1 contains only 1331 unplaced scaffolds with a total length of 58.3 Mb, which is much less than ARS1 (29,875 of 340.6 Mb). Based on the position of the centromeric regions that we identified below (Fig. 1), the orientations of 14 chromosomes (chr2, 3, 4, 7, 10, 12, 14, 15, 17, 18, 23, 26, 27, and 28) were reversed compared to ARS1, but agreed with those in the CHIR_2.0 genome assembly.

**Table 1:** Comparison of chromosome lengths, gaps and telomere lengths between the Saanen_v1 and ARS1 genome assemblies

Chr	Saanen_v1			ARS1		
	Ungapped length (bp)	Gap number	Telomere length (bp)	Ungapped length (bp)	Gap number	Telomere length (bp)
1	157,026,289	12	9940	157,403,278	10	0
2	136,815,899	4	0	136,510,747	8	0
3	122,007,170	4	0	120,037,984	11	0
4	121,353,546	4	0	120,733,315	7	0
5	119,111,638	5	0	119,019,111	12	0
6	118,192,308	2	13,717	117,637,248	15	0
7	108,721,103	4	11,502	108,433,436	8	5318
8	114,262,317	5	13,891	112,671,558	11	0
9	92,377,540	1	7926	91,568,381	8	5195
10	102,383,334	7	14,353	101,087,335	9	24,448
11	106,922,721	12	16,286	106,224,777	9	0
12	88,153,514	6	18,575	87,276,782	18	0
13	83,177,721	5	6611	83,032,465	9	0
14	94,829,756	1	11,734	94,672,533	8	0
15	83,141,260	4	11,585	81,900,668	12	0
16	80,964,695	4	2424	79,367,392	12	0
17	73,078,104	0	10,066	71,136,580	9	0
18	67,211,768	4	4767	67,274,927	20	4698
19	62,759,990	3	12,832	62,516,200	10	2180
20	72,063,028	4	14,488	71,782,370	5	2421
21	70,281,929	3	5029	69,423,070	13	0
22	60,513,250	2	5029	60,280,842	8	0
23	52,518,510	6	18,806	48,866,424	5	2053
24	62,595,921	1	8188	62,310,016	2	0
25	43,127,748	3	14,990	42,858,159	14	0
26	51,538,664	1	16,511	51,421,353	8	0
27	44,839,895	0	0	44,708,984	2	0
28	44,621,055	0	0	44,672,302	0	0
29	51,316,256	1	18,141	51,332,371	13	0
X	142,335,304	37	5497	115,936,137	214	0
Y	9,603,805	24	5310	–	–	–
Sum	2,637,846,038	169	278,198	2,582,096,745	490	46,313

The ungapped length was calculated for each sequence by excluding gap regions which were represented by Ns

The assembly size of Saanen_v1 (2.69 Gb) was smaller than that of ARS1 (2.92 Gb) but relatively close to that of the previous goat reference genome CHIR_2.0 (2.72 Gb) and the estimated genome size of 2.72 Gb by kmer analysis (see Methods) and (see Additional file 2: Figure S1). We note that Saanen_v1 harbors longer chromosome sequences (2.63 Gb) and a slightly higher repeat content (1.22 Gb) than ARS1 (2.58 Gb and 1.20 Gb, respectively) (see Additional file 3: Table S4). When we mapped the high coverage (40×) Illumina short-reads sequencing data against Saanen_v1 and ARS1, the average genome coverages were similar for both assemblies; ARS1 possessed a much larger proportion of unplaced scaffolds (218.7 Mb) than Saanen_v1 (26.3 Mb) with almost no coverage (depth < 1 in 1-kb window) (see Additional file 2: Figure S3). Compared to ARS1, Saanen_v1 is closer to the estimated physical size of the goat genome, indicating that many of the unplaced scaffolds in ARS1 should be used with caution.

### Quality assessment

As a measure of genome completeness and to define the chromosome ends, we examined centromeric and telomeric repeats across the chromosomes. All of the autosomes and the X chromosome of goats are acrocentric. Considering that the highly repeated mega base centromere and telomere regions on the short arms of chromosomes are unlikely to be fully resolved, we would expect centromeric and telomeric repeats at the proximal and distal ends, respectively, for a nearly complete acrocentric chromosome. Indeed, we detected telomeric and centromeric repeats at the expected locations on 24 and 27 chromosomes (Fig. 1), respectively. Remarkably, we identified centromeric and telomeric repeats at the proximal and distal ends of approximately two-thirds (20/29) of the autosomes, indicating that the assemblies of these chromosomes were close to being complete. For the X chromosome, we identified the centromeric region at 135.13–135.20 Mb, but an additional centromeric signal was found at 8.1–37.3 kb which may indicate an ancient centromere position prior to chromosomal rearrangements. In contrast, telomeres were observed only on seven, six and five chromosomes of the ARS1, sheep (Oar_rambouillet_v1.0) and cattle (ARS-UCD1.2) reference genome assemblies (see Additional file 3: Table S5). Moreover, compared with ARS1 and other assemblies, Saanen_v1 harbors more and longer centromeric repeats per chromosome (see Additional file 3: Table S6).

**Fig. 2:**
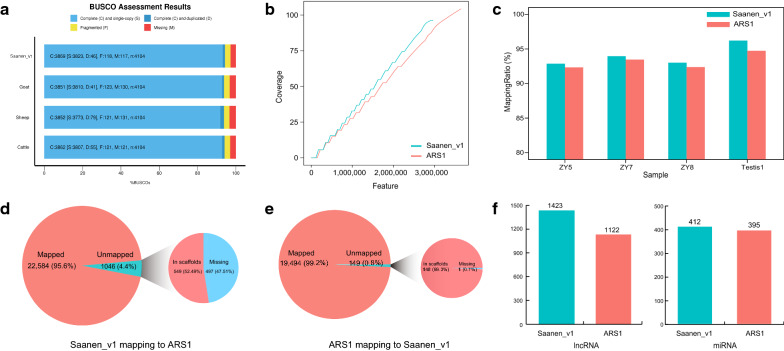
Quality assessment of the Saanen goat genome assembly. **a** BUSCO assessment results of four ruminant genome assemblies. Saanen_v1 is compared with the reference genome of goat (ARS1, GCA_001704415.1), sheep (Oar_rambouillet_v1.0, GCA_002742125.1) and cattle (ARS-UCD1.2, GCA_002263795.2). **b** Feature response curves computed for Saanen_v1 and ARS1. **c** Comparison of mapping rate for four Iso-seq data. **d** Gene annotation mapping from Saanen_v1 to ARS1. **e** Gene annotation mapping from ARS1 to Saanen_v1. **f** Number of annotated lncRNAs and miRNAs in Saanen_v1 and ARS1

Saanen_v1 was then assessed for completeness by Benchmarking Universal Single-Copy Orthologs (BUSCO) [49] analysis. The results suggest that Saanen_v1 is highly complete with a BUSCO score of 94.3%, compared with the previous goat genome assembly (ARS1, 93.8%) and other reference genomes including sheep (Oar_rambouillet_v1.0, 93.8%) and cattle (ARS-UCD1.2, 94.1%) (Fig. 2a) and (see Additional file 2: Figure S4). The FRC_align tool [54] was used to identify erroneous regions in the alignment file and to plot a feature response curve to show discrepancies between the two assemblies (Fig. 2b). Compared with ARS1, Saanen_v1 displayed a smaller number of COMPR_PE and STRECH_PE, representing a lower level of erroneous sequence compressions and expansions (see Additional file 3: Table S7). Although the numbers of HIGH COV PE and HIGH NORM COV PE were larger in Saanen_v1 than in ARS1 (which might indicate some collapsed repetitive regions), ARS1 contains a much larger number of LOW_COV_PE and LOW_NORM_COV_PE features than Saanen_v1, representing regions with low read coverage. The same short-read alignments were also used to estimate the quality value (QV) of the assemblies with Saanen_v1 scoring 30.0 and ARS1 34.6. The lower QV for Saanen_v1 could be partially attributed to the fact that ARS1 was selected from one panel of 96 US goats with higher homozygosity [13]. Indeed, we observed only slightly lower QV for Saanen_v1 than ARS1 using three additional Illumina short-reads datasets from Asian (25.4 vs. 25.9), European 25.8 vs. 26.2 and African (25.6 vs. 26.1) individuals (see Methods), which suggested that the two assemblies displayed comparable base accuracy.

We also compared the mappability of sequence reads between ARS1 and Saanen_v1 based on whole-genome sequencing data from five additional unrelated individuals (10–40×). The short-read data were mapped to ARS1 and Saanen_v1. The results showed similar mapping rates for Saanen_v1 and ARS1 (98.43% vs. 98.41%) (see Additional file 3: Table S8). We also observed a slight increase in the mapping rate of RNA-seq data for Saanen_v1 than for ARS1 (97.01% vs. 96.81%; P < 0.05, two-tailed paired t-test) (see Additional file 3: Table S9). A noticeable increase in the mapping rate was observed for long-read Iso-seq data from four tissue samples (94.0% vs. 93.2%; P < 0.05, two-tailed paired t-test) (Fig. 2c) and (see Additional file 3: Table S10).

To evaluate the quality of the assembly, we further performed gene annotation projection between Saanen_v1 and ARS1. To do this, we reciprocally mapped gene annotations derived from one assembly to the other using the Liftoff tool [62], which is a robust gene mapping tool for genome assemblies of the same species. Generally, if an assembly shows a lower mapped ratio than its counterpart, then we would expect it to be more complete and accurate, since it contains gene annotations that cannot be projected on the other. In this study, using a combination of multiple approaches (see Methods), we annotated 23,630 protein-coding genes in Saanen_v1. In total, 95.6% of the genes could be mapped to chromosomes in ARS1, while 549 genes were mapped to unplaced scaffolds and 497 genes were unmapped (Fig. 2d). In contrast, when mapping gene annotations from ARS1 to Saanen_v1, approximately 99.2% could be mapped to chromosomes while 148 genes were mapped to unplaced scaffolds and only one gene remained unmapped (Fig. 2e).

The long non-coding RNA (lncRNA) and miRNA genes were also annotated on Saanen_v1 and ARS1, using the same RNA-seq and miRNA-seq data, respectively (see Methods). As expected, more lncRNA and miRNA genes were found on chromosomes of Saanen_v1 than on those of ARS1 (lncRNA: 1,423 vs. 1,122; miRNA: 412 vs.395) (Fig. 2f).

### Comparison of the sequence collinearity between the Saanen_v1 and ARS1 genome assemblies

We performed whole-genome alignment of Saanen_v1 with ARS1 using MUMmer [38] and found good collinearity between the two assemblies, except for the X chromosome (see Additional file 2: Figure S5), as many assembly errors were identified in the ARS1 X chromosome. Then, structural variations were detected using Assemblytics [39] based on the whole-genome alignments. In total, 16,714 structural variations (> 50 bp) with a total length of 11.7 Mb were identified, including 5887 deletions, 5181 insertions, 1228 repeat contractions, 1026 repeat expansions, 1723 tandem contractions, and 1669 tandem expansions (see Additional file 3: Table S11) and (see Additional file 2: Figure S6). Then, we found that 6190 of the structural variations intersected with various functional genomic elements including coding and untranslated regions (see Additional file 4: Table S12).

**Fig. 3:**
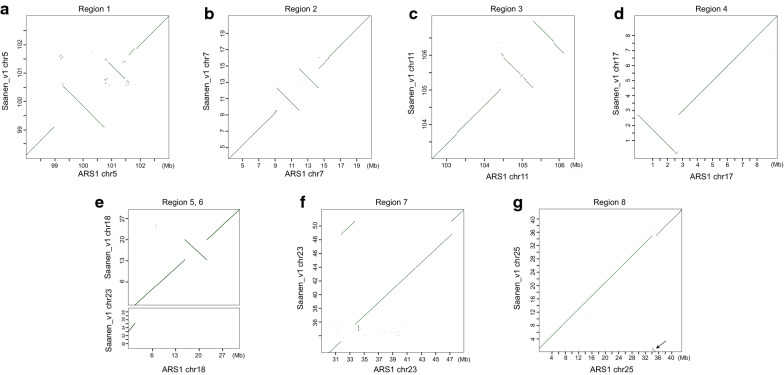
Sequence alignment uncovers the eight putative large misassemblies by comparing the Saanen_v1 and ARS1 assemblies. **a**–**d** Represent inverted Regions 1 to 4 as shown in Table 2; **e** The top panel represents Region 5 which is inverted and the bottom panel represents Region 6 which is incorrectly mapped to chr18 on ARS1; **f** represents Region 7 which is incorrectly placed on chr23; **g** represents Region 8 which is incorrectly placed on chr25. For each panel, the X axis represents the region in ARS1 and the Y axis represents the corresponding region in Saanen_v1. The diagonal lines represent the matching alignments between the two sequences whereas those that are perpendicular to diagonals represent inverted regions and those that are parallel to diagonals represent incorrectly placed regions

Remarkably, we found eight large inconsistent regions (> 1 Mb each) between Saanen_v1 and ARS1 that were located on autosomes, spanning 24.0 Mb (Fig. 3 and Table 2). The longest region spanned approximately 6.3 Mb on chr18 of ARS1, and it was inverted compared with Saanen_v1. The sequenced Hi-C data for each assembly were mapped to the corresponding assembly to assess whether the Hi-C contact matrix supported the assembled sequence. Our manual curation showed that these regions were likely due to assembly errors in ARS1, as seven of them could be confirmed by the Hi-C contact matrix, which showed signals of discrepancy in ARS1 but not in Saanen_v1 (see Additional file 2: Figure S7). The other inconsistent region (Region 3) was supported by collinearity of the corresponding regions of Saanen_v1 and the sheep reference genome (Oar_rambouillet_v1.0) (see Additional file 2: Figure S8). Most of the assembly errors could also be confirmed by collinearity between Saanen_v1 and CHIR_2.0 in the corresponding regions (see Additional file 2: Figure S9).

**Table 2:** Eight large putative assembly errors in autosomes of the ARS1 assembly as compared with the Saanen_v1 assembly

Name	Position in Saanen_v1	Position in ARS1	Length (bp)	Description
Region 1	5:99,084,275–101,783,455	5:98,973,162–101,785,323	2,812,161	Inverted
Region 2	7:9,511,868–14,628,369	7:9,353,868–13,740,007	4,386,139	Inverted
Region 3	11:103,733,339–106,912,333	11:103,232,270–106,224,990	2,992,720	Inverted
Region 4	17:61,267–2,700,348	17:0–2,620,773	2,620,773	Inverted
Region 5	18:14,220,403–20,470,517	18:16,211,458–22,469,539	6,258,081	Inverted
Region 6	23:33,110,782–35,437,285	18:0–1,987,427	1,987,427	Incorrectly placed
Region 7	23:48,920,741–50,832,698	23:32,018,169–33,927,923	1,909,754	Incorrectly placed
Region 8	25:40,139–1,085,452	25:34,034,345–35,098,278	1,063,933	Incorrectly placed

**Fig. 4:**
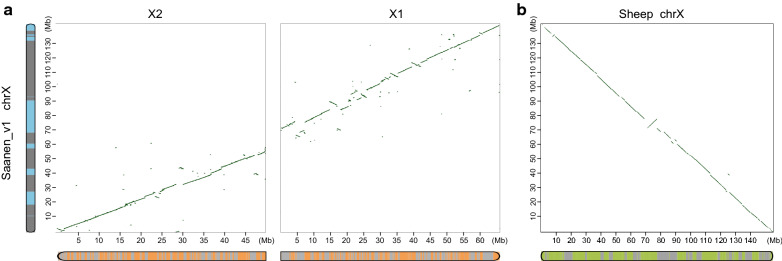
Improvement in the assembly continuity of X chromosome. **a** Sequence alignment between the X chromosomes of Saanen_v1 (Y axis), and ARS1 (X1 and X2 in Y axis). X1 and X2 are the two scaffolds of the X chromosome in the ARS1 assembly. **b** Sequence alignment between the X chromosomes of Saanen_v1 (Y axis) and sheep reference genome (Oar_rambouillet_v1.0, X axis). Each color block on the chromosome ideogram represents contigs

The X chromosome assembly in ARS1 contains two scaffolds with 319 gaps, summing up to 115.9 Mb. In contrast, the X chromosome in Saanen_v1 is assembled into one scaffold with only 37 gaps. Furthermore, it spans 142.4 Mb, which is close to its expected size of 150 Mb [72]. This new X chromosome assembly is also more accurate, as evidenced by better collinearity with that of the sheep (Oar_rambouillet_v1.0) and CHIR_2.0 assemblies (Fig. 4).

### Y chromosome assembly and estimation of the substitution rate

**Fig. 5:**
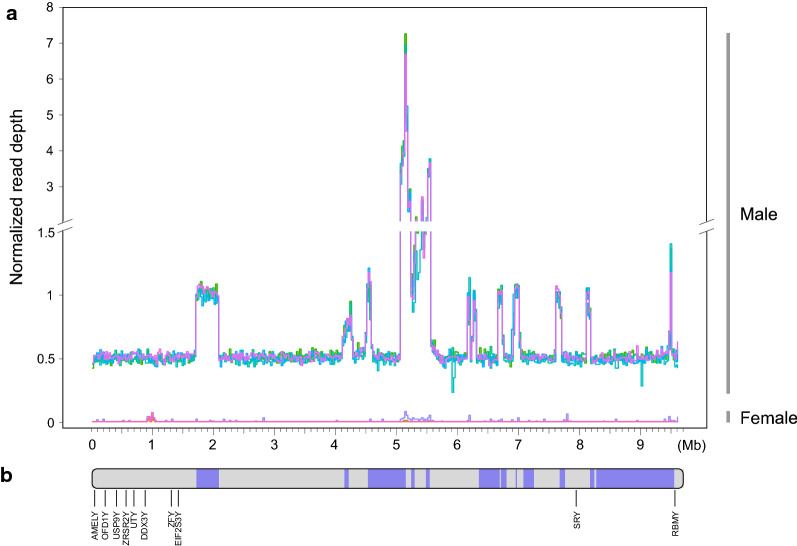
Generation of the first goat chromosome Y assembly. **a** The normalized read depth of whole-genome sequencing data on the goat Y chromosome for males and females. Whole-genome sequencing data from five bucks and five does were used. Read depth was then calculated in 1-kb window with a 500-bp step size and normalized to the autosomal average. Those peaks with high depth reflected ampliconic regions with high coverage. **b** Sequence alignment of the goat chromosome Y assembly and Y-linked scaffolds from ARS1. Each color block on the chromosome ideogram represents contigs

In this study, we generated the first draft assembly of the goat Y chromosome with a single scaffold of 9.6 Mb, representing the male-specific region. From the Hi-C heatmap, the identified Y chromosome scaffold and X chromosome reside on opposite sides of the pseudoautosomal regions (PAR), agreeing with the fact that the Y chromosome and X chromosome shared the homologous PAR (see Additional file 2: Figure S10). In addition, to confirm the male-specific regions, whole-genome sequencing data from five males and five females were mapped to our new assembly, displaying exclusive coverage only with reads from male samples (Fig. 5a). Then, we annotated 10 known single-copy genes (*AMELY*, *OFD1Y*, *USP9Y*, *ZRSR2Y, UTY*, *DDX3Y*, *ZFY*, *EIF2S3Y*, *SRY*, and *RMBY*) (Fig. 5b) and three multi-copy gene families (*HSFY*, *ZNF280AY*, and *ZNF280BY*) by comparative gene annotation using Y chromosome genes from sheep [73] and cattle [74]. Furthermore, we searched all available published *Capra* Y-linked amplicons from the NCBI nucleotide database and found nine entries. All of them could be aligned to our Y chromosome assembly with 100% coverage and more than 98% identity, except for one entry (AY082491 with 94.6% identity) (see Additional file 3: Table S13).

We aligned the goat Y chromosome with those of three ruminant species (cattle, yak and sheep) that are publicly available (see Methods). In addition, to obtain conservative estimates of substitution rates, we retained only alignment blocks for which all four species were present, thus largely restricting our analysis to the X-degenerate regions of approximately 645.1 kb. From the multispecies alignments, the goat Y chromosome was highly similar in sequence to the ovine Y (96.1%) and less similar to the cattle Y (91.7%) and yak Y (91.8%), which is in agreement with the phylogeny of these species.

**Fig. 6:**
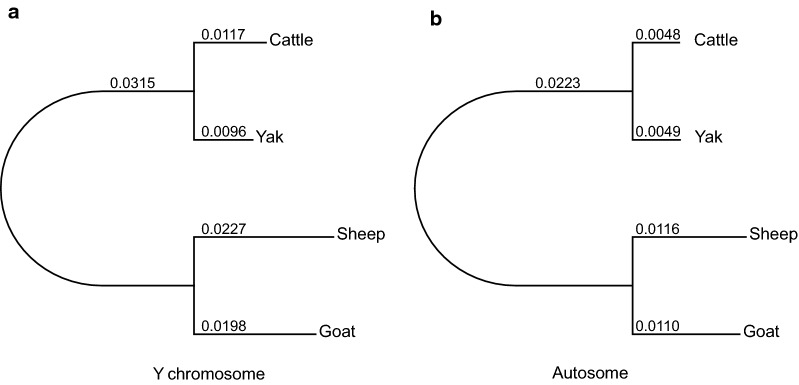
Phylogenetic tree of nucleotide sequences. **a** Y chromosome. **b** Autosomes. Branch lengths (substitutions per 100 sites) were estimated from multispecies alignment blocks including the four species (cattle, yak, sheep and goat)

We also estimated the substitution rates of the Y chromosomes and the autosomes. A higher substitution rate was observed for the Y chromosome than for the autosomes for each branch (Fig. 6), which is potentially due to male mutation bias [75]. The male-to-female mutation rate ratio (α_m_) in goats was estimated to be 3.5 based on its Y-to-autosomal substitution rate (see Methods). Furthermore, a similar Y-to-autosomal substitution rate was found for goats (1.55) and sheep (1.56), implying that the evolutionary rate of Caprini species has remained stable after their divergence ~ 5.85 million years ago. Moreover, the Y-to-autosomal substitution rate seems to be close to that of cattle (1.58) but much higher than that of yak (1.26), which might reflect their different evolutionary histories and deserves to be further investigated.

### SNP chip probes mapped to Saanen_v1

**Fig. 7:**
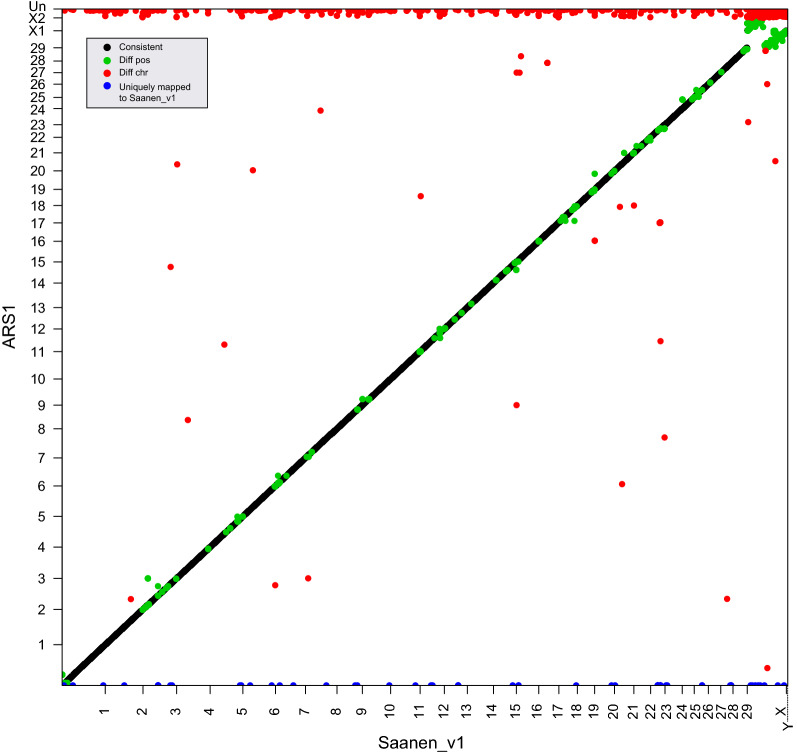
Probes from the GoatSNP50 chip showing SNP positional discrepancies between Saanen_v1 (X axis) and ARS1 (Y axis)

Incorrect SNP position assignment affects genotype imputation and linkage disequilibrium, therefore leading to false coverage and reduced power of genome-wide association analysis. To identify SNP positional discrepancies, all SNP probes from the commercial GoatSNP50 chip were mapped to the Saanen_v1 and ARS1 assemblies. Among the 53,347 SNPs on this chip, the majority could be mapped to both assemblies, and 95.4% were positionally consistent. Compared with their position on ARS1, 4.4% (2364/53347) of the SNP probes displayed positional discrepancies (Fig. 7), including 908 on different chromosomes (diff chr), 1395 showing changed rank order within the same chromosome (diff pos), and 69 uniquely mapped to Saanen_v1 (see Additional file 5: Table S14). Notably, 91.7% (833/908) of those belonging to “diff chr” were due to the position adjustments from scaffolds in ARS1 to chromosomes of Saanen_v1. For those in the category of “diff pos”, 42.7% (597/1395) of the SNP probes were found on the X chromosome, and another 33.6% (469/1395) resided in the eight large assembly error regions that were identified in this study. Therefore, our results suggest that most of the SNP positional discrepancies are likely due to sequence improvement in Saanen_v1 enabling us to assign the correct positions for a considerable number of SNP probes from the GoatSNP50 chip.

In addition, we identified regions of the genome that were poorly represented by markers in the goat GoatSNP50 chip. In total, we found nine large regions (> 1 Mb each) (see Additional file 3: Table S15) that were completely devoid of SNPs on Saanen_v1, of which the longest region spanned 4.9 Mb on chromosome X. It should be noted that, when examining their positions on CHIR_1.0 from which the goat GoatSNP50 chip was designed, 26 poorly represented regions were found, spanning 150 to 202 kb without any large gap regions longer than 1 Mb. Therefore, the large underrepresented regions that we found here were most likely due to improved sequence resolution of the goat genome and thus should be taken into consideration for updating the goat SNP chip.

## Discussion

In this study, we assembled a highly continuous genome assembly of goat by leveraging large contig lengths from PacBio long reads and Hi-C with high sequencing depth. The ARS1 assembly was generated from one individual with high levels of homozygosity, to minimize heterozygous regions in the genome and thereby reduce difficulties in de novo assembly [76, 77]. Nevertheless, the continuous improvement in long-read sequencing technology has greatly alleviated previous limitations [78, 79], enabling us to generate a high-quality reference genome for Saanen dairy goats.

Saanen_v1 with its contig N50 of 46.2 Mb and 169 gaps surpasses the current goat genome and compares favorably with other livestock reference genome sequences. Most of the chromosomes of Saanen_v1 are longer than those of ARS1 indicating that more sequences have been assembled into chromosomes. Only a few chromosomes were found to have proper telomeric and centromeric signals in the current goat reference genome (ARS1) as well as in the sheep references (Oar_rambouillet_v1.0) and cattle ones (ARS-UCD1.2), which were compared in this study. The reference genomes of buffalo and pig were also reported to contain very few telomeric repeats, which were not directly compared here due to their different karyotypes. The capture of telomeric and centromeric signals at the opposite ends of most chromosomes (Fig. 1) provides compelling evidence that Saanen_v1 likely surpasses most reference genome assemblies in terms of sequence completeness and continuity.

The improvement in Saanen_v1 is not restricted to continuity but also includes improvement in sequence composition by providing more complete sequences and correcting putative errors in ARS1. On the one hand, we reported eight large assembly errors in ARS1 by whole-genome alignment, one of which (Region 6) was also found in our previous goat pan-genome study [17]. The other large assembly errors that we reported here could not be verified, since they are caused by inversions, whereas our previous goat pan-genome study mainly focused on the non-reference sequences from ARS1. On the other hand, 86.6% of the non-reference sequences reported in our goat pan-genome study that were due to assembly errors were found in Saanen_v1, whereas the rest were not found in Saanen_v1, (see Additional file 1: Supplementary method) for the detailed verification of previously reported assembly errors in ARS1, probably representing ARS1-specific sequences.

The most significant improvements in Saanen_v1 concerned the sex chromosomes. The X chromosome sequence that we generated in our study (spanning 142.4 Mb) was much longer than that of ARS1 (two scaffolds, 115.9 Mb in total) and CHIR_2.0 (131.6 Mb) [1]. We also generated the first goat Y chromosome assembly, spanning 9.6 Mb, comparable with the ovine Y chromosome assembly that we recently generated using Nanopore sequencing. However, the current long-read sequencing from either Nanopore or PacBio technologies, as well as genome assembly algorithms still cannot resolve the complex regions of the Y chromosome. We anticipate that the application of chromosome flow sorting for the Y chromosome [80] and the generation of highly accurate long reads (PacBio HiFi reads) [81] to resolve complex regions will eventually enable us to obtain the complete sequence of the goat Y chromosome. Nevertheless, the single-copy regions were successfully recovered with only a few contigs which is adequate for SNP discovery in domestic goats to trace the evolution and diversity of paternal lineages. Furthermore, we propose that the X and Y chromosome sequences generated from Saanen_v1 could be incorporated into the current goat reference genome by replacing the corresponding scaffolds (see Additional file 6: Table S16) to facilitate downstream genomic and biological research investigations.

With the availability of the first Capra Y chromosome assembly, we were able to estimate and compare the substitution rate among four ruminant lineages (goat, sheep, cattle, and yak). We observed higher substitution rates for the Y chromosome than for autosomes across the four lineages, suggesting that the Caprini and Bovini species are subjected to male-driven molecular evolution [82]. This male mutation bias has also been observed in other mammalian species, including primates and rodents [83], which could be attributed to the relative excess of cell divisions in the male germline compared with the female germline [75]. We also found a similar substitution rate for the Y chromosome of goat and sheep which shared a common ancestor 5.85 million years ago, implying that the evolution rates in Caprini species are similar. In contrast, a previous study showed that the Y-to-autosomal substitution rate for humans is much lower than that for chimpanzee, although they diverged 6.6 million years ago [84]. Furthermore, we reported a male-to-female mutation rate (α_m_) of about 3.5 for goats, which is in agreement with previous reports [82]. We also found that the α_m_ is smaller in goats than in primates but is larger than in rodents [75], which is probably a result of different evolutionary histories [85].

Our study implied that the quality of reference genome assemblies will continue to improve with advances in single molecular sequencing technologies and assembly methods and reduced sequencing costs. For example, the first complete human X chromosome from telomere to telomere has been achieved using a combination of Nanopore and PacBio sequencing technology [18]. Eventually, the gapless and accurate genome assemblies with high-quality sequences will be available for goat and other livestock species and allow us to discover the full spectrum of SNPs associated with quantitative trait loci (QTL). The higher resolution of SNPs will increase the accuracy of imputation and inference of linkage disequilibrium between specific alleles of SNPs and QTL, thus improving the reliability of genomic prediction in genomic selection programs. In addition, many studies have pointed out that a single genome is inadequate for a variety of reasons, such as lack of diversity [86–88] or inherent bias towards the reference genome. Therefore, breed reference assemblies will be required, especially for cosmopolitan breeds, such as the Saanen dairy goat that we sequenced in this study. With the availability of abundant de novo assemblies, it is expected that the single linear reference genome will be replaced by a new paradigm—a graph genome that could better reflect the diversity of animal species.

## Conclusions

We generated a high-quality de novo genome assembly (Saanen_v1) from a Saanen buck using PacBio long-read sequencing and Hi-C. This new Saanen_v1 assembly displays appreciable improvements in sequence completeness and continuity as compared with the current goat reference genome (ARS1). Notably, it includes a continuous X chromosome sequence and the first goat Y chromosome scaffold. Saanen_v1 will facilitate genetic diversity studies and implementations of GWAS and genomic selection in goats.

## Supplementary Information

**Additional file 1.** Supplementary methods for Hi-C library preparation and verification of previously reported assembly errors in ARS1

**Additional file 2: Figure S1.** 17-mer count distribution for the goat genome size estimation. The 17-mer count distribution was used to estimate genome size. Note that the peaks around the depths of 36, 70 and 138 represent the heterozygous, homozygous and repeated Kmers, respectively. **Figure S2.** Comparison of assembly quality among various reference genome assemblies. The gap number and contig N50 of seven species were compared. The gap number and contig N50 (Mb) for each assembly are shown in the brackets. **Figure S3.** Read depth across chromosomes (top right panel) and unplaced scaffolds (main panel). The read depth of chromosomes and unplaced scaffolds was compared between Saanen_v1 and ARS1. The whole genome sequencing data of a Yunnan black goat (~ 40×) are mapped to Saanen_v1 and ARS1. The read depth was calculated in 1-kb non-overlapping window. **Figure S4.** Venn diagram showing the intersection of identified genes among the 4104 single-copy orthologs in mammalia_obd9 database for BUSCO analysis. The intersection of identified genes from BUSCO analysis is shown for the four genome assemblies. **Figure S5.** Whole-genome alignment between Saanen_v1 and ARS1. The collinearity between Saanen_v1 and ARS1 is shown by whole-genome alignment. The Y chromosome and scaffolds of the two assemblies were excluded from the alignments. **Figure S6.** Structural variations detected in Saanen_v1 as compared with ARS1. The figure was generated by Assemblytics, displaying the summary statistics of structural variations. **Figure S7.** Hi-C contact matrix of ARS1 supports that the discrepancy between the alignments is likely due to assembly errors in ARS1. The assembly errors in ARS1 was evidenced by the Hi-C contact matrix. For each putative error region, the Hi-C heatmaps from ARS1 (left panel) and Saanen_v1 (right panel) were shown with the arrows indicating the discordant signals potentially caused by incorrect assembly. **Figure S8.** Alignment of the Saanen_v1 assembly and the sheep genome for the regions surrounding chr11:103,733,339–106,912,333 bp. The agreement between Saanen_v1 and sheep suggested that the corresponding region in ARS1 was incorrect. **Figure S9.** Sequence alignment between Saanen_v1 and CHIR_2.0 for the eight putative regions with assembly errors. Most of the assembly errors could be confirmed by collinearity between Saanen_v1 and CHIR_2.0 in the corresponding regions. **Figure S10.** The Hi-C heatmap shows that the putative Y and X chromosomes reside on the proximal and distal ends of PAR. We used Hi-C heatmap to infer X and Y chromosomes by locating their shared PAR region.

**Additional file 3: Table S1.** The publicly available miRNA-seq data used for miRNA annotation. We downloaded publicly available miRNA-seq data to annotate miRNA genes. **Table S2.** Summary of raw reads from PacBio sequencing. The data summarized the reads counts and length of PacBio sequencing. **Table S3.** Comparison of the basic statistics of the Saanen_v1 and ARS1 assemblies. The assembly length and continuity were compared between Saanen_v1 and ARS1. **Table S4.** Comparison of the repeat content of Saanen_v1 with ARS1. The total repeat content of Saanen_v1 was slightly higher than ARS1. The unplaced scaffolds from each assembly were not included for comparison. **Table S5.** Telomere signals identified in each assembly. Telomeres were found on 27 chromosomes of Saanen_v1 compared with 7, 6 and 5 chromosomes of ARS1, sheep (Oar_rambouillet_v1.0) and cattle (ARS-UCD1.2), respectively. **Table S6.** Centromere signals identified in each assembly. Saanen_v1 harbors more and longer centromeric repeats per chromosome than the other three assemblies of the reference genome for goat (ARS1), sheep (Oar_rambouillet_v1.0) and cattle (ARS-UCD1.2). **Table S7.** Structural inconsistencies when comparing Saanen_v1 and ARS1 assemblies. Various categories of structural inconsistencies were compared between Saanen_v1 and ARS1 using the FRC_align tool. **Table S8.** Mapping ratio of whole-genome sequencing data. Four whole-genome sequencing data of domestic goats were aligned to Saanen_v1 and ARS1 to compare the mapping ratio. **Table S9.** Mapping ratio of RNA-seq data. Nine RNA-seq datasets were aligned to Saanen_v1 and ARS1 to compare the mapping ratio. **Table S10.** Mapping ratio of Iso-seq data. Four Iso-seq datasets were aligned to Saanen_v1 and ARS1 to compare the mapping ratio. **Table S11.** Summary of structural variations in Saanen_v1 as compared with ARS1. The data represented the count and total length of structural variations in Saaenen_v1 as compared with ARS1. **Table S13.** Blast alignment of previously reported goat Y chromosome amplicons to the Y chromosome assembly included in Saanen_v1. The previously reported goat Y chromosome amplicons could be aligned to our generated Y chromosome assembly, implying the validity of our sequence. **Table S15.** Information on the large gap regions (> 1 Mb) between two adjacent SNP probes. The data presented seven large gap regions which were not covered by SNP probes.

**Additional file 4: Table S12.** Gene annotation of identified structural variations between Saanen_v1 and ARS1. The structural variations between Saanen_v1 and ARS1 were annotated using ANNOVAR.

**Additional file 5: Table S14.** List of probes showing SNP positional discrepancy. The data listed the probes that showed SNP positional discrepancy in Saanen_v1 as compared with ARS1.

**Additional file 6: Table S16.** Y chromosome-linked scaffolds in ARS1 that we suggest to be replaced by our Y chromosome scaffold from Saanen_v1. The Y chromosome-linked scaffolds in ARS1 were listed which could be replaced by our new Y chromosome scaffold from Saanen_v1 to facilitate downstream analysis.

## Data Availability

Sequences and metadata generated in this work are publicly available. All the data including PacBio sequencing, WGS and Hi-C are deposited at the Sequence Read Archive (https://www.ncbi.nlm.nih.gov/sra) under accession number PRJNA613503. The Saanen goat genome is available in GenBank with accession number GCA_015443085.1. Two Iso-seq datasets used in the current study are not publicly released but are available from the corresponding author on reasonable request.
