# Supplementary material for: A near complete genome for goat genetic and genomic research
Source: Genet Sel Evol. 2021 Sep 10;53:74. doi: 10.1186/s12711-021-00668-5 (PMC8434745; doi:10.1186/s12711-021-00668-5)
Supplement: Supplementary file 1 — Additional file 1. Supplementary methods for Hi-C library preparation and verification of previously reported assembly errors in ARS1 [file 12711_2021_668_MOESM1_ESM.docx]

**Additional file 1**

**Supplementary methods**

**Hi-C library preparation**

Fresh blood sample was used for Hi-C library construction and sequencing. Three milliliters of blood were used for cross-link with1% (final concentration) fresh formaldehyde following by 0.2 M (final concentration) of Glycine quenched for 5 min. Then the cross-linked cells were lysed in lysis buffer (10 mM Tris-HCI (pH 8.0), 10 mM NaCl, 0.2% NP40, and complete protease inhibitors (Roche Mannheim, Germany)). Extracted nuclei were re-suspended in 150 µl of 0.1% SDS and then incubated at 65 °C for 10 min, then SDS molecules were quenched by adding 120 μl water and 30 μl 10% Triton X-100 following by incubation at 37 °C for 15 min. DNA from nuclei was digested by adding 30 μl 10x NEB buffer 2.1 (50 mM NaCl, 10 mM Tris-HCl, 10 mM MgCl2, 100 μg/ml BSA, pH 7.9) and 150 U of MboI, and then incubated at 37 °C overnight. On the second day, the Mbol enzyme was inactivated at 65 °C for 20 min first and then cohesive ends were filled by adding 1 μl of 10 mM dTTP, 1μl of 10 mM dATP, 1 μl of 10 mM dGTP, 2 μl of 5mM biotin-14-dCTP, 14 μl water and 4 μl (40 U) Klenow, and incubated at 37 °C for 2 h. The proximity ligation was carried out at 16 °C for 4 h, by adding 663 μl water,120 μl 10x blunt-end ligation buffer (300 mM Tris-HCl, 100 mM MgCl2, 100 mM DTT, 1 mM ATP, pH 7.8), 100 μl 10% Triton X-100 and 20 U T4 DNA ligase. After ligation, the cross-linking was reversed by 200 μg/mL proteinase K at 65°C overnight.

The QIAamp DNA Mini Kit (Qiagen, Hilden, Germany) was used to purify DNA according to manufacturers’ instructions. Purified DNA was sheared to a length of approximately 300 bp. Point ligation junctions were pulled down with Dynabeads® MyOne™ Streptavidin C1 (Thermofisher Cambridge, UK). The Hi-C library for Illumina sequencing was prepped by NEBNext® Ultra™ II DNA library Prep Kit for Illumina (NEB, Ipswich, USA) according to manufacturers’ instructions. The sequencing of the final Hi-C library was performed on the Illumina HiSeq X Ten platform (Illumina, San Diego, USA) in the paired-end mode of 150 bp.

**Verification of previously reported assembly errors in ARS1**

In our previous goat pan-genome study, we used “assembly-versus-assembly” approach to discover pan-sequences (non-reference sequences) which were absent in reference genome, including true structural variations and potential assembly errors in ARS1. However, the final call sets did not include other types of structural variations such as inversions. In contrast, the eight putative large assembly errors were mainly due to inversions and, thus, could not be identified from our previous study. Therefore, we mainly verified whether the proportion of pan-sequences due to assembly errors from our goat pan-genome study could be found in Saanen_v1.

To find the pan-sequences, all whole genome sequencing data from the individual used to generate ARS1 were aligned to the reference genome (ARS1 plus our previous reported pan-sequences) by using the BWA MEM v0.7.13-r1126 [31]. Those pan-sequences with ≥80% coverage were considered as assembly errors (4.5 Mb). Then these pan-sequences were aligned to Saanen_v1 using the BLAST v2.2.31 [66]. We finally identified 3.9 Mb non-reference sequences with identity of at least 90% and coverage of at least 50%, indicating that the most (86.6%) of the assembly errors were corrected in Saanen_v1. The remaining sequences which cannot be found in Saanen_v1 might represent ARS1-specific sequences.
