# Supplementary material for: A near complete genome for goat genetic and genomic research
Source: Genet Sel Evol. 2021 Sep 10;53:74. doi: 10.1186/s12711-021-00668-5 (PMC8434745; doi:10.1186/s12711-021-00668-5)
Supplement: Supplementary file 2 — Additional file 2: Figure S1. 17-mer count distribution for the goat genome size estimation. The 17-mer count distribution was used to estimate genome size. Note that the peaks around the depths of 36, 70 and 138 represent the heterozygous, homozygous and repeated Kmers, respectively. Figure S2. Comparison of assembly quality among various reference genome assemblies. The gap number and contig N50 of seven species were compared. The gap number and contig N50 (Mb) for each assembly are shown in the brackets. Figure S3. Read depth across chromosomes (top right panel) and unplaced scaffolds (main panel). The read depth of chromosomes and unplaced scaffolds was compared between Saanen_v1 and ARS1. The whole genome sequencing data of a Yunnan black goat (~ 40×) are mapped to Saanen_v1 and ARS1. The read depth was calculated in 1-kb non-overlapping window. Figure S4. Venn diagram showing the intersection of identified genes among the 4104 single-copy orthologs in mammalia_obd9 database for BUSCO analysis. The intersection of identified genes from BUSCO analysis is shown for the four genome assemblies. Figure S5. Whole-genome alignment between Saanen_v1 and ARS1. The collinearity between Saanen_v1 and ARS1 is shown by whole-genome alignment. The Y chromosome and scaffolds of the two assemblies were excluded from the alignments. Figure S6. Structural variations detected in Saanen_v1 as compared with ARS1. The figure was generated by Assemblytics, displaying the summary statistics of structural variations. Figure S7. Hi-C contact matrix of ARS1 supports that the discrepancy between the alignments is likely due to assembly errors in ARS1. The assembly errors in ARS1 was evidenced by the Hi-C contact matrix. For each putative error region, the Hi-C heatmaps from ARS1 (left panel) and Saanen_v1 (right panel) were shown with the arrows indicating the discordant signals potentially caused by incorrect assembly. Figure S8. Alignment of the Saanen_v1 assembly and the sheep genome f [file 12711_2021_668_MOESM2_ESM.docx]

**Additional materials**

**A near complete genome for goat genetic and genomic research**

**This file includes:**

**Additional Fig S1-S10**

**
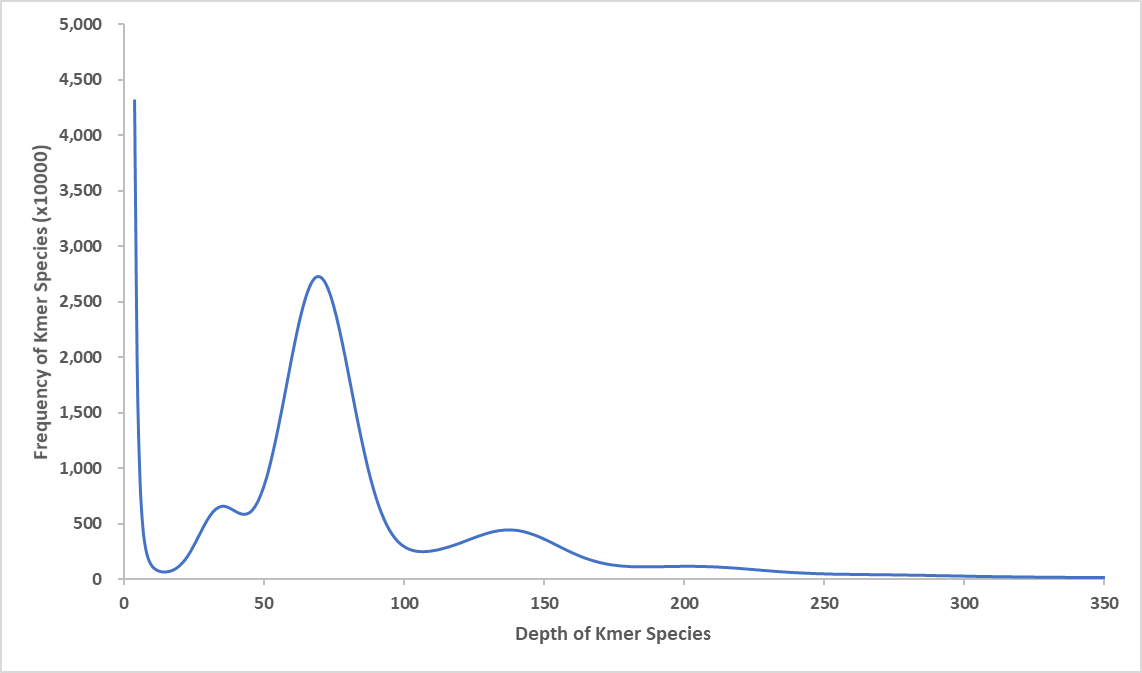
**

**Fig. S1 17-mer count distribution for the goat genome size estimation.** Note that the peaks around the depths of 36, 70 and 138 represent the heterozygous, homozygous and repeated kmers, respectively.


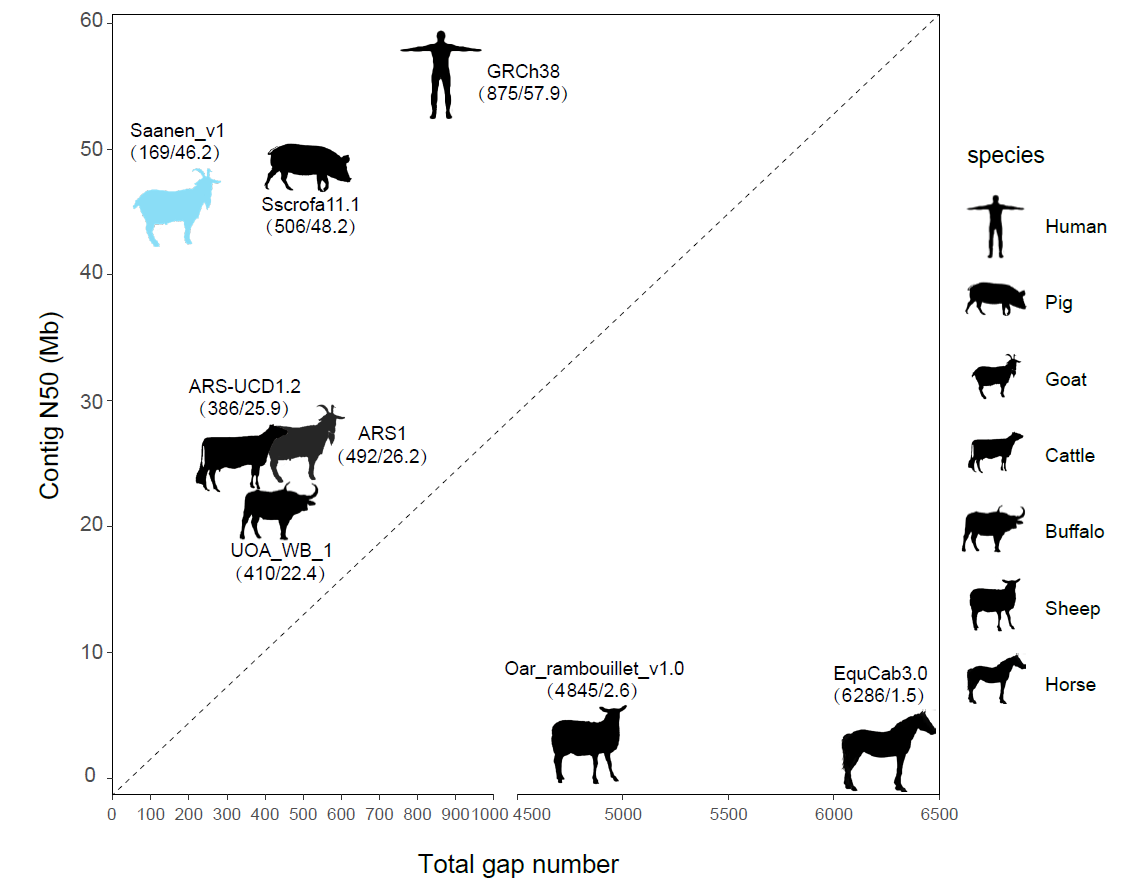


**Fig. S2 Comparison of assembly quality among various reference genome assemblies.** The gap number and contig N50 (Mb) for each assembly are shown in the brackets.


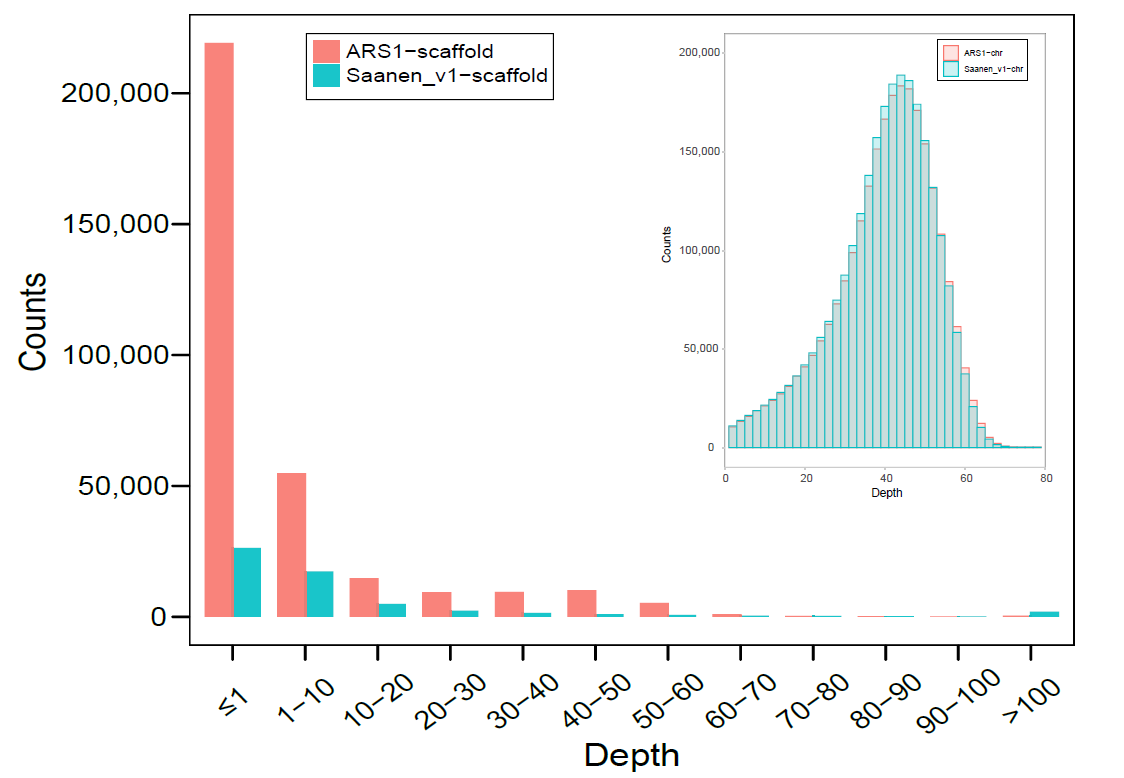


**Fig. S3 Read depth across chromosomes (top right panel) and unplaced scaffolds (main panel).** The whole genome sequencing data of a Yunnan black goat (~40×) are mapped to Saanen_v1 and ARS1. The read depth was calculated in 1 Kb non-overlapping window.


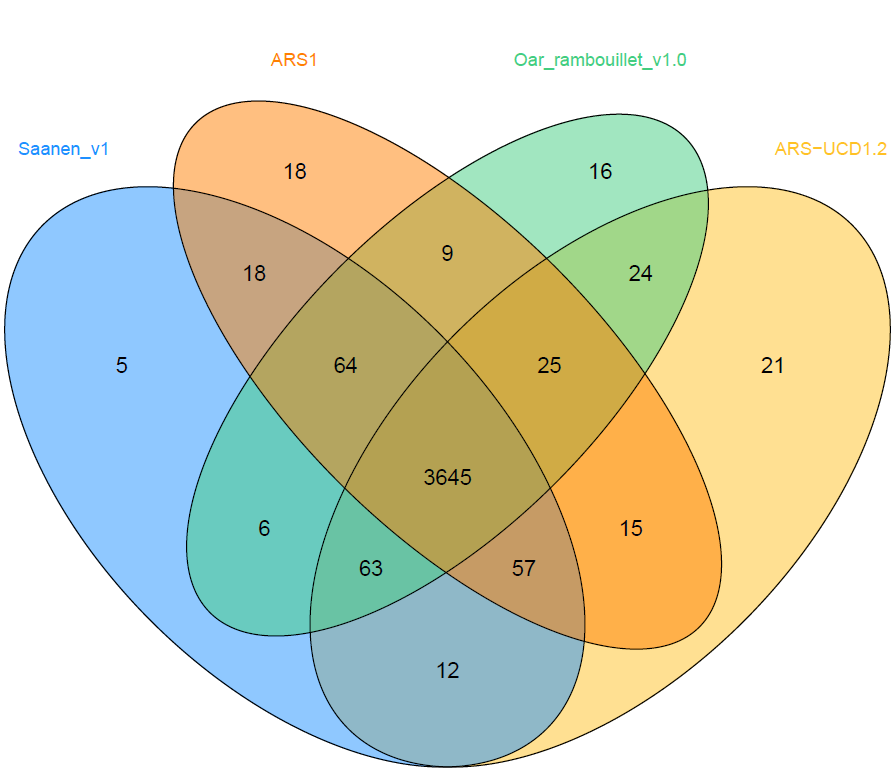


**Fig. S4 Venn diagram showing the intersection of identified genes among the 4104 single-copy orthologs in mammalia_obd9 database for BUSCO analysis.**


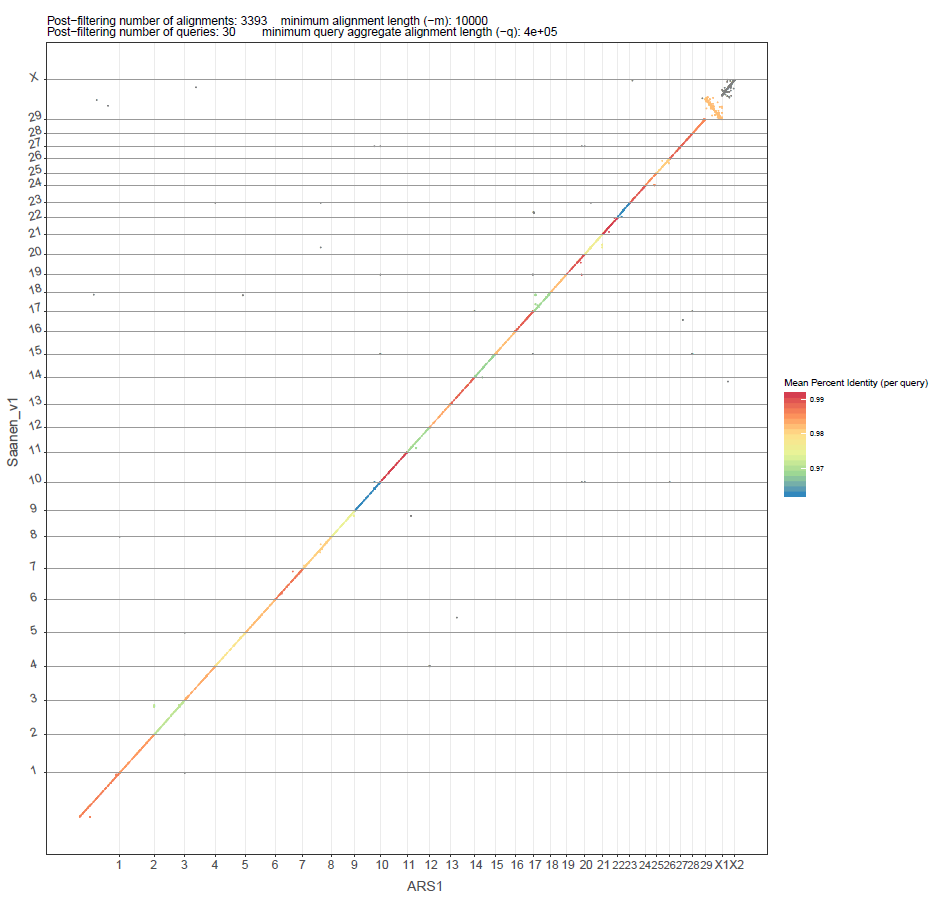


**Fig. S5 Whole genome alignment between Saanen_v1 and ARS1.** Y chromosome and scaffolds of the two assemblies were excluded from the alignments.


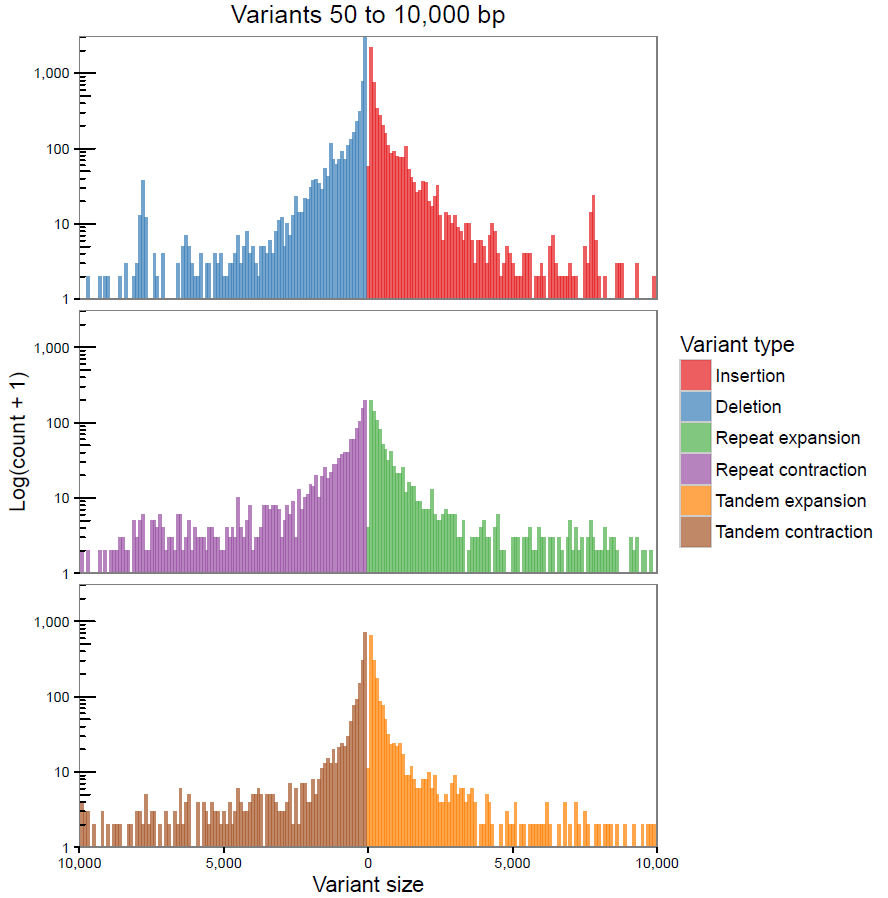


**Fig. S6 Structural variations detected in Saanen_v1 as compared with ARS1.**


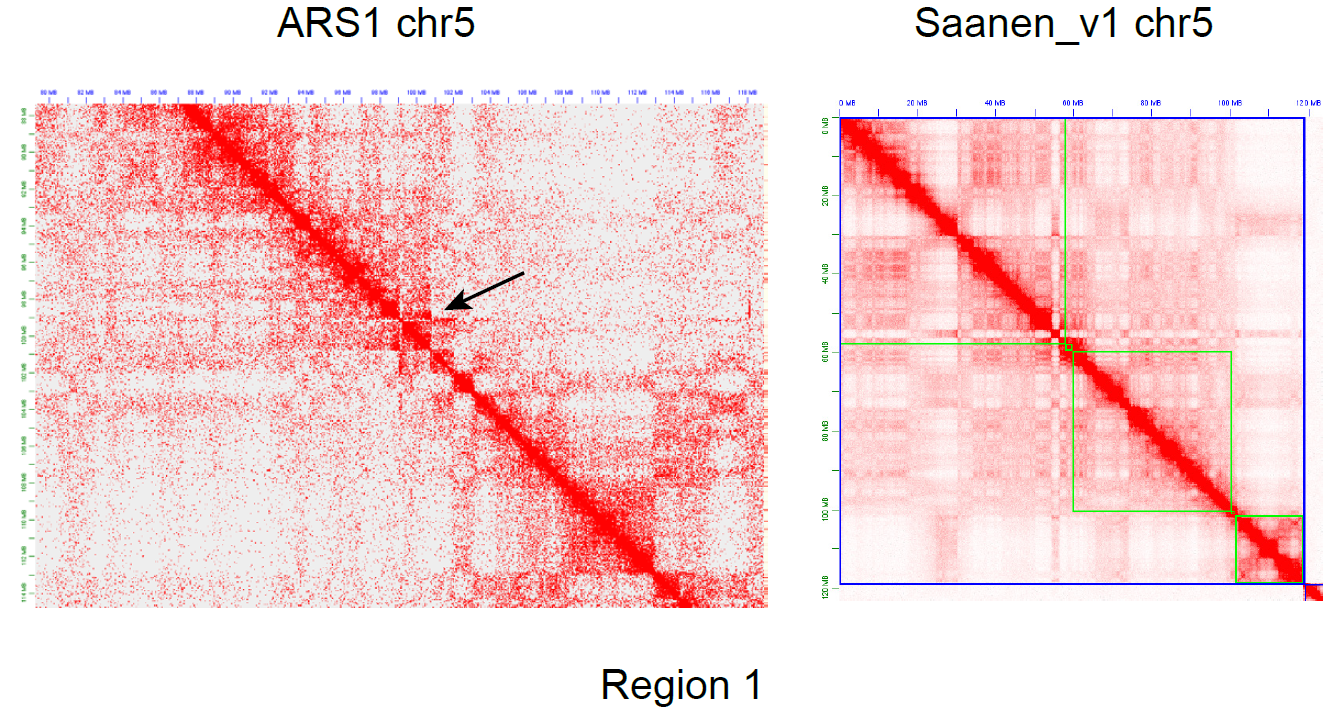

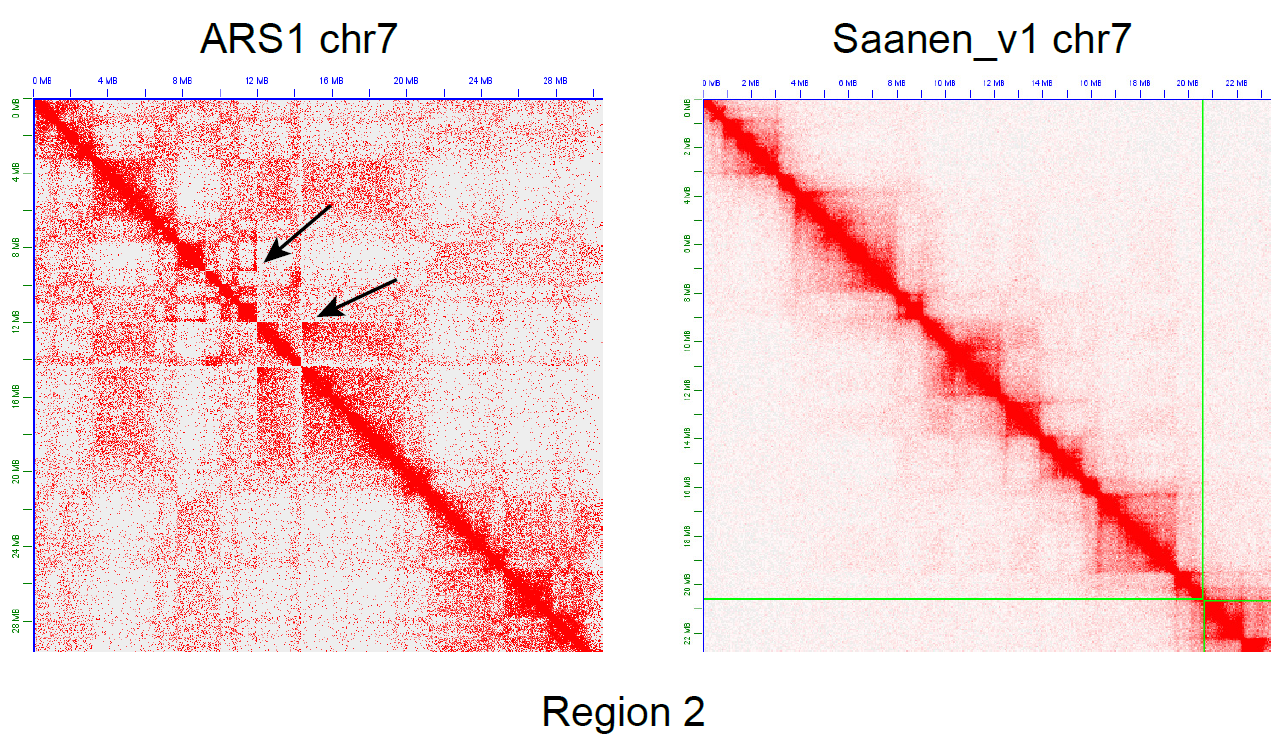


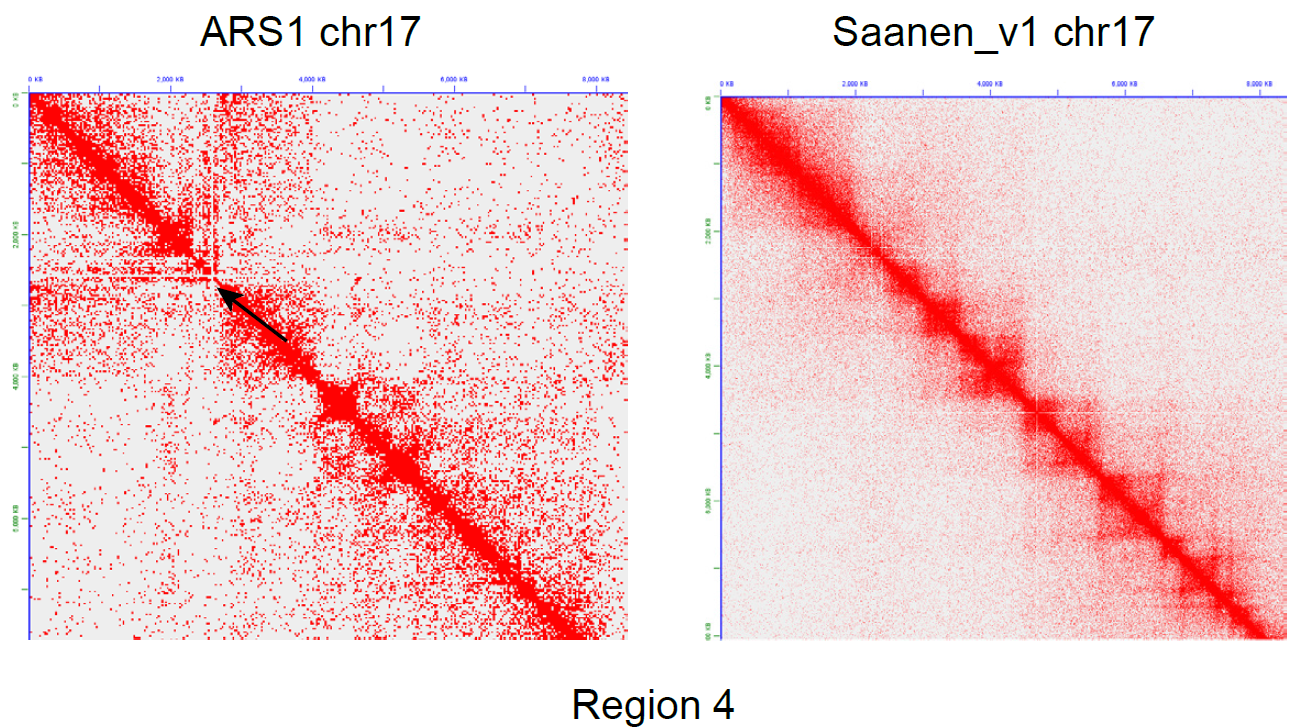


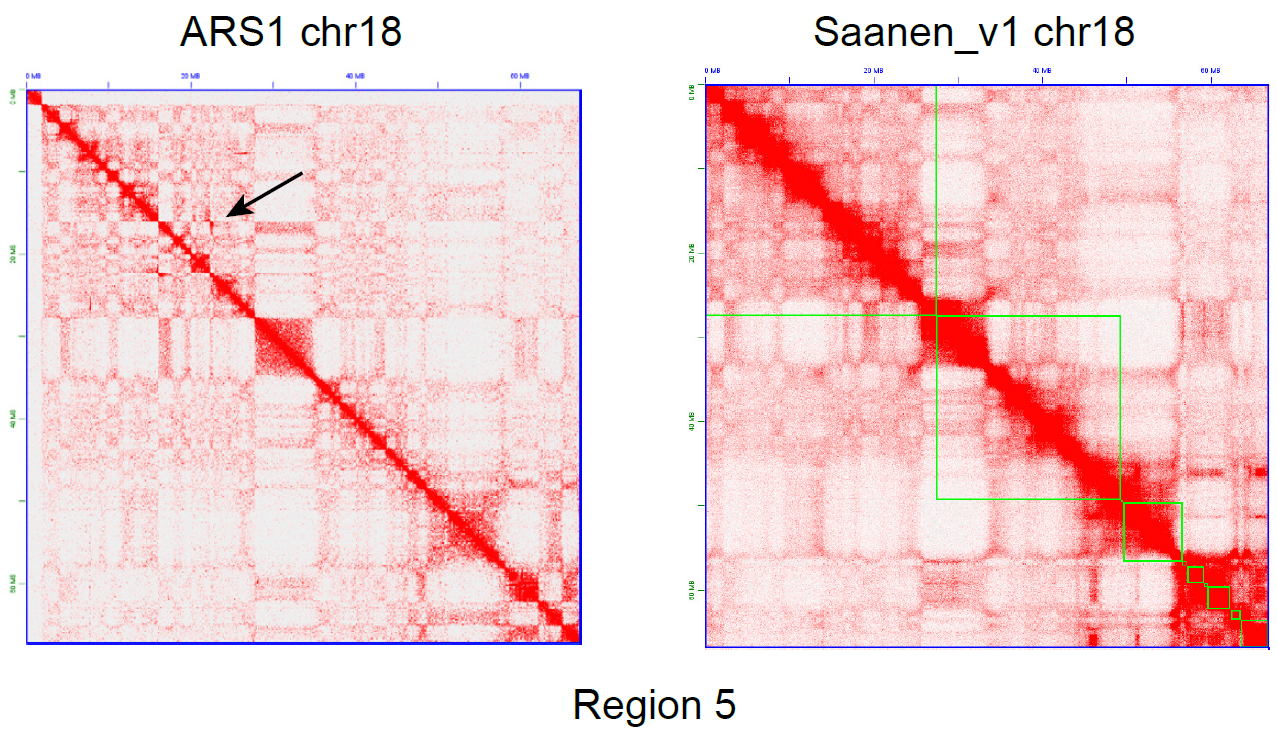


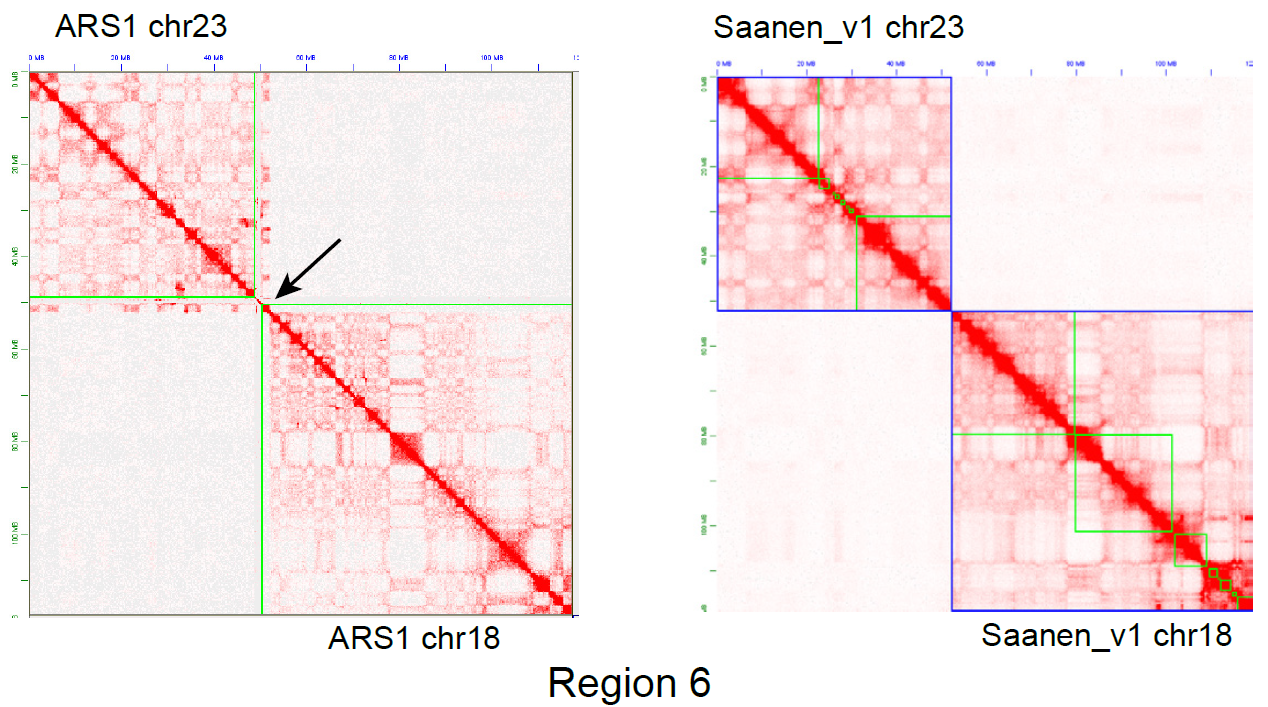


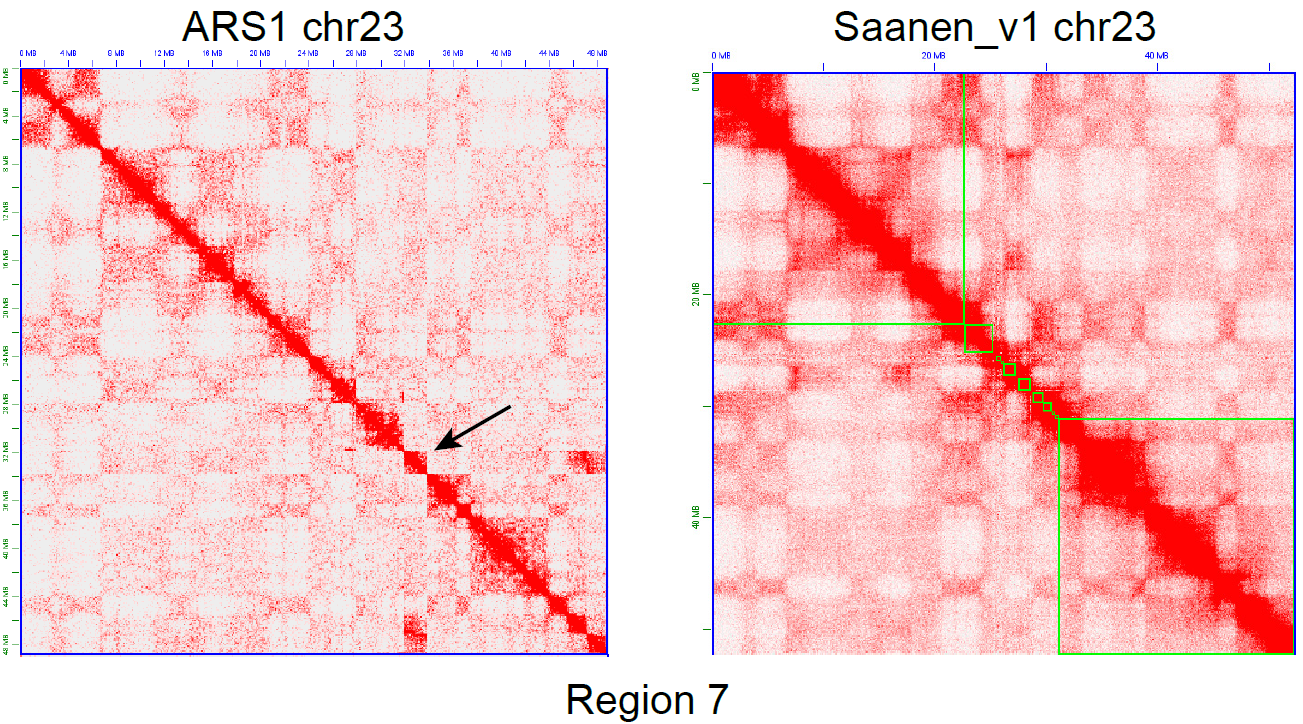


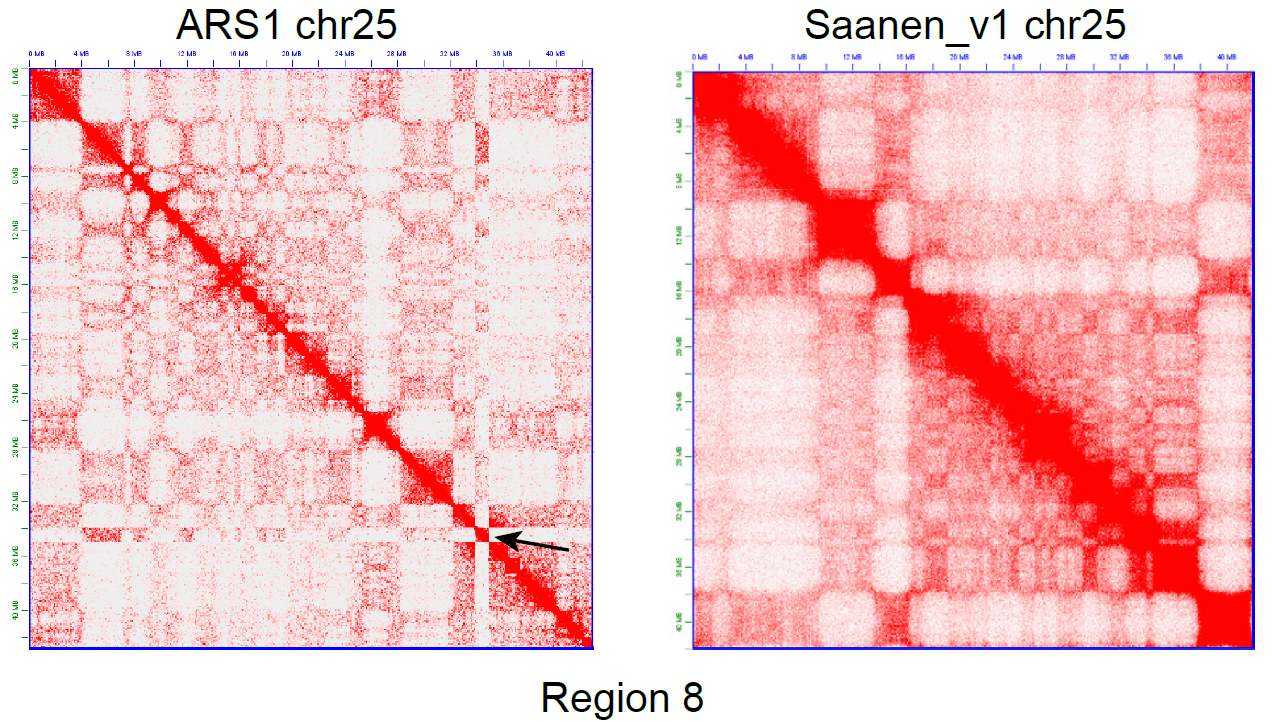


**Fig. S7 Hi-C contact matrix of ARS1 support that the discrepancy between the alignments is likely due to assembly errors in ARS1.** For each putative error region, the Hi-C heatmaps from ARS1 (left panel) and Saanen_v1 (right panel) were shown with the arrows indicating the discordant signals potentially caused by incorrect assembly.

**
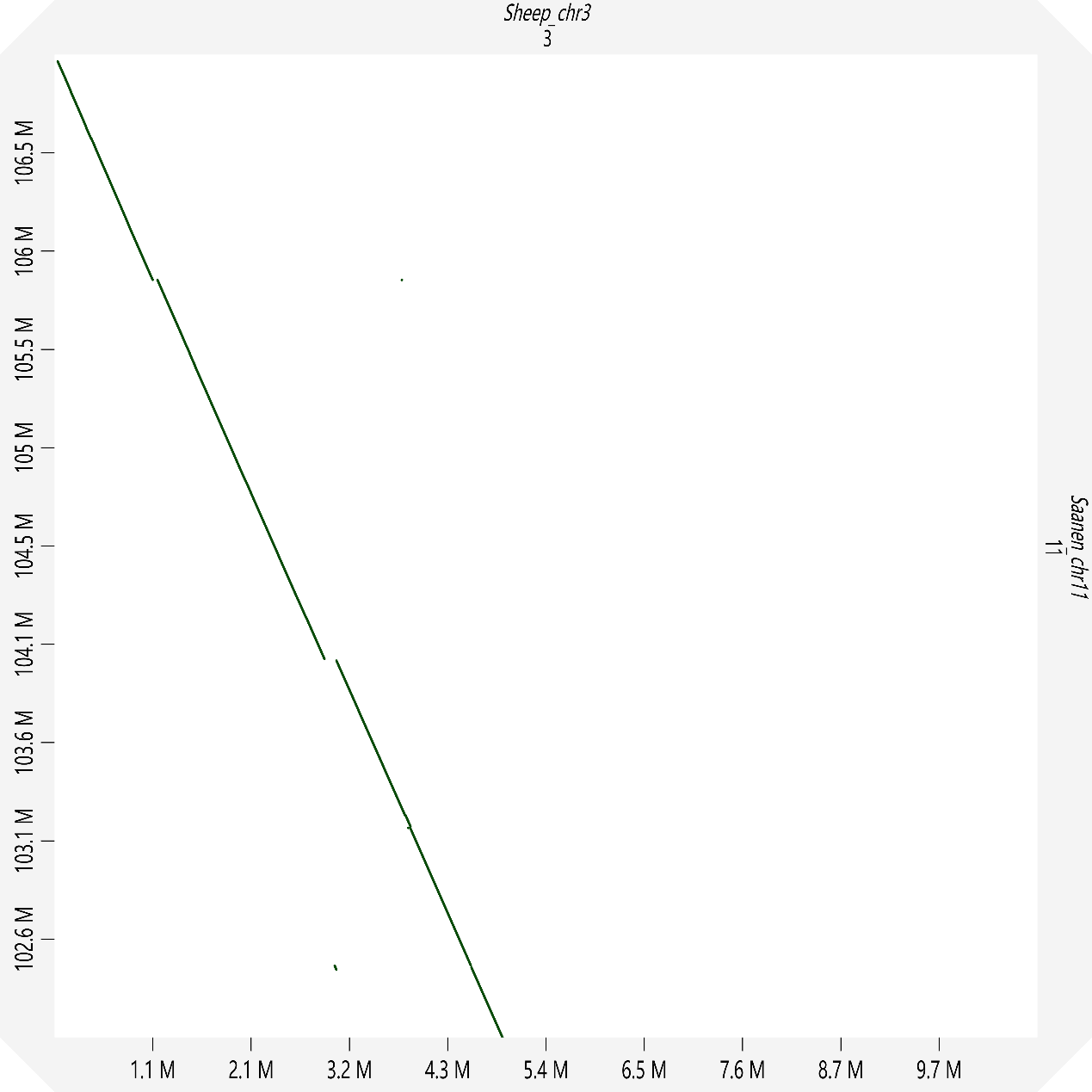
**

**Fig. S8 Alignment of Saanen_v1 and sheep genome for the regions surrounding chr11:103,733,339-106,912,333.**


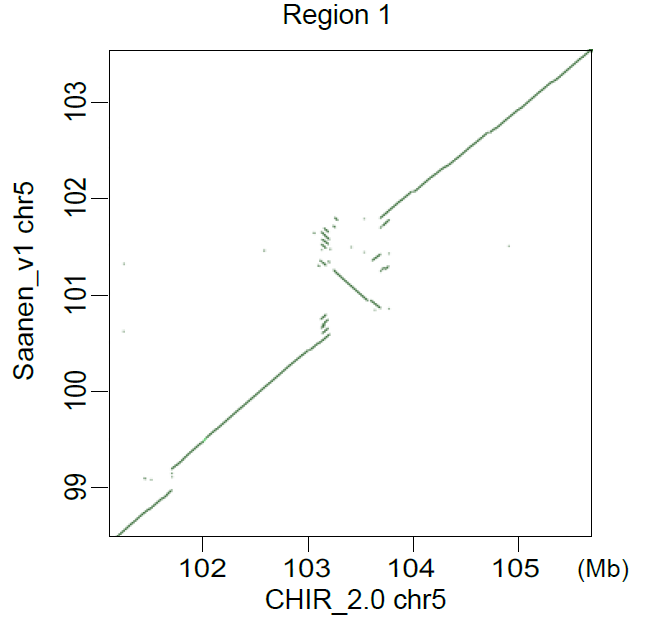

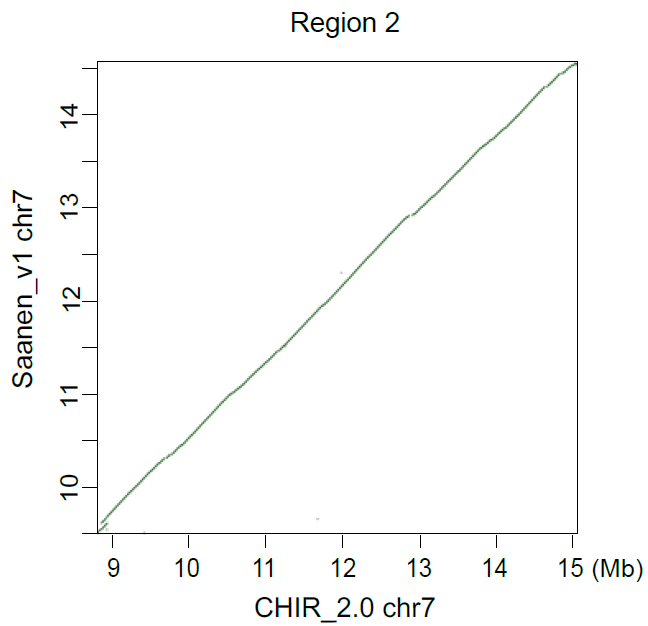


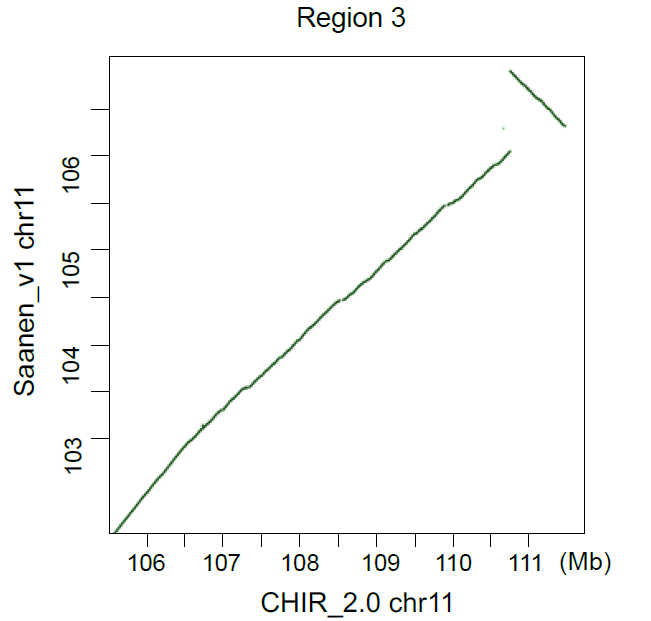

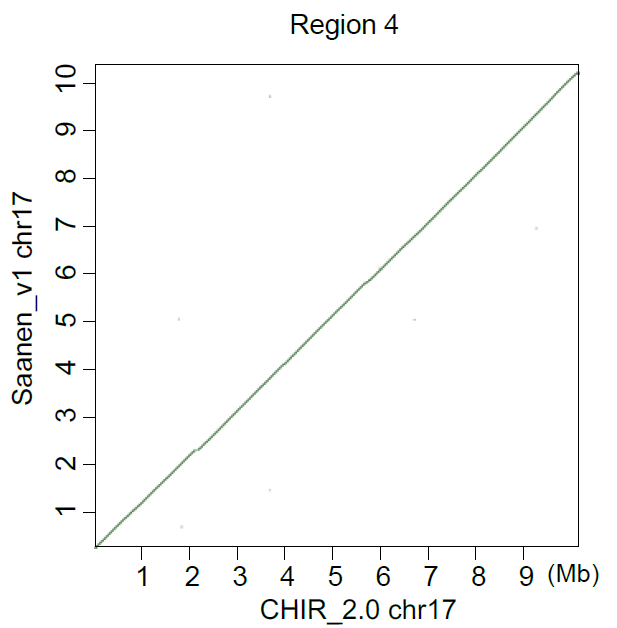


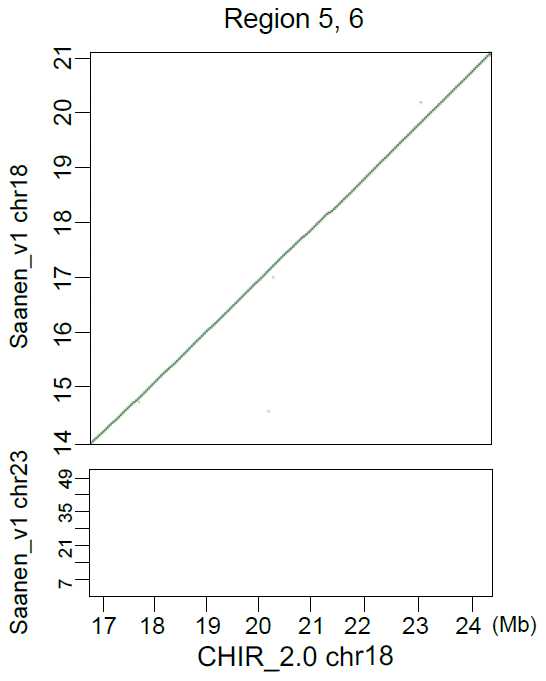

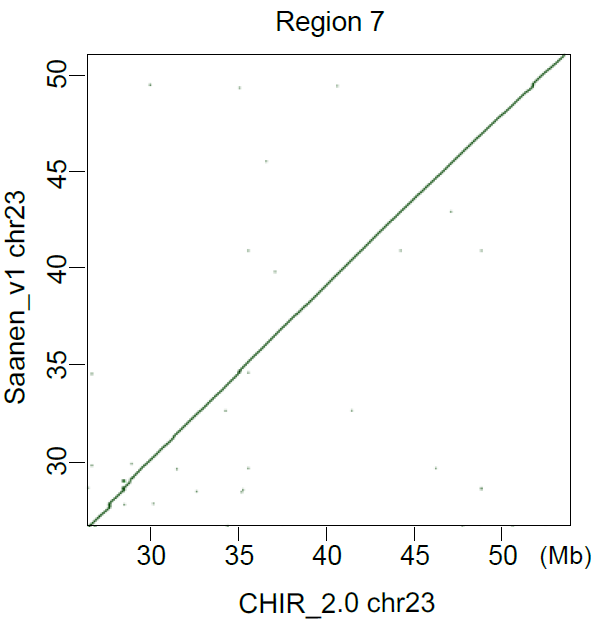


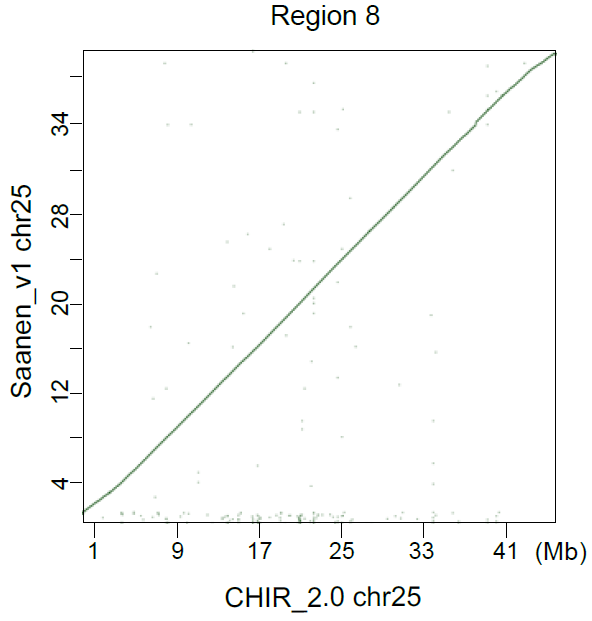


**Fig. S9 Sequence alignment between Saanen_v1 and CHIR_2.0 for the eight putative assembly error regions.**


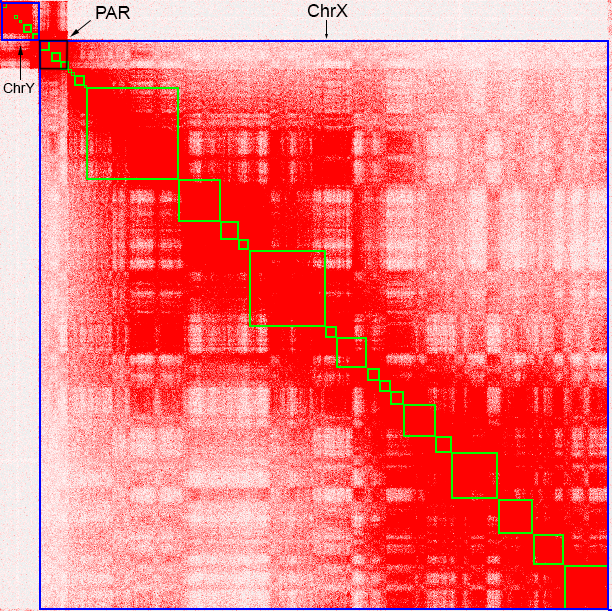


**Fig. S10 The Hi-C heatmap shows that the putative Y and X chromosomes reside on the proximal and distal ends of PAR.**
