# Supplementary material for: A near complete genome for goat genetic and genomic research
Source: Genet Sel Evol. 2021 Sep 10;53:74. doi: 10.1186/s12711-021-00668-5 (PMC8434745; doi:10.1186/s12711-021-00668-5)
Supplement: Supplementary file 3 — Additional file 3: Table S1. The publicly available miRNA-seq data used for miRNA annotation. We downloaded publicly available miRNA-seq data to annotate miRNA genes. Table S2. Summary of raw reads from PacBio sequencing. The data summarized the reads counts and length of PacBio sequencing. Table S3. Comparison of the basic statistics of the Saanen_v1 and ARS1 assemblies. The assembly length and continuity were compared between Saanen_v1 and ARS1. Table S4. Comparison of the repeat content of Saanen_v1 with ARS1. The total repeat content of Saanen_v1 was slightly higher than ARS1. The unplaced scaffolds from each assembly were not included for comparison. Table S5. Telomere signals identified in each assembly. Telomeres were found on 27 chromosomes of Saanen_v1 compared with 7, 6 and 5 chromosomes of ARS1, sheep (Oar_rambouillet_v1.0) and cattle (ARS-UCD1.2), respectively. Table S6. Centromere signals identified in each assembly. Saanen_v1 harbors more and longer centromeric repeats per chromosome than the other three assemblies of the reference genome for goat (ARS1), sheep (Oar_rambouillet_v1.0) and cattle (ARS-UCD1.2). Table S7. Structural inconsistencies when comparing Saanen_v1 and ARS1 assemblies. Various categories of structural inconsistencies were compared between Saanen_v1 and ARS1 using the FRC_align tool. Table S8. Mapping ratio of whole-genome sequencing data. Four whole-genome sequencing data of domestic goats were aligned to Saanen_v1 and ARS1 to compare the mapping ratio. Table S9. Mapping ratio of RNA-seq data. Nine RNA-seq datasets were aligned to Saanen_v1 and ARS1 to compare the mapping ratio. Table S10. Mapping ratio of Iso-seq data. Four Iso-seq datasets were aligned to Saanen_v1 and ARS1 to compare the mapping ratio. Table S11. Summary of structural variations in Saanen_v1 as compared with ARS1. The data represented the count and total length of structural variations in Saaenen_v1 as compared with ARS1. Table S13. Blast alignment of previously [file 12711_2021_668_MOESM3_ESM.docx]

**Additional file 3**

**Table S1. The publicly available miRNA-seq data used for miRNA annotation**

| **Sample Name** | **Read length (bp)** | **Total bases (Mb)** | **Population** | **SRA** | **Tissue** |
| --- | --- | --- | --- | --- | --- |
| GSM3579835 | 50 | 593.3 | Chuandong White Goat | SRP182057 | heart |
| GSM3579841 | 50 | 577.7 | Chuandong White Goat | SRP182057 | heart |
| GSM3579847 | 50 | 586.1 | Chuandong White Goat | SRP182057 | heart |
| GSM3579832 | 50 | 568.1 | Chuandong White Goat | SRP182057 | liver |
| GSM3579838 | 50 | 564.8 | Chuandong White Goat | SRP182057 | liver |
| GSM3579844 | 50 | 557.1 | Chuandong White Goat | SRP182057 | liver |
| GSM3579836 | 50 | 541.6 | Chuandong White Goat | SRP182057 | muscle |
| GSM3579842 | 50 | 539 | Chuandong White Goat | SRP182057 | muscle |
| GSM3579848 | 50 | 557.7 | Chuandong White Goat | SRP182057 | muscle |

**Table S2. Summary of raw reads from PacBio sequencing**

| Library | Total bases (bp) | Subread number | Average subread length (bp) | Subread length N50 |
| --- | --- | --- | --- | --- |
| lib1 | 101,881,444,550 | 5,205,710 | 19,571 | 31,244 |
| lib2 | 93,599,784,091 | 4,770,817 | 19,619 | 31,764 |
| lib3 | 132,205,513,590 | 7,170,183 | 18,438 | 29,815 |
| total | 327,686,742,231 | 17,146,710 |  |  |

**Table S3. Basic statistics of Saanen_v1 assembly as compared with ARS1**

|  | ARS1 | Saanen_v1 |
| --- | --- | --- |
| Total assembly length (bp) | 2,922,813,246 | 2,696,218,280 |
| Total ungapped length | 2,922,775,059 | 2,696,156,580 |
| Gap number | 773 | 169 |
| Number of contigs | 30,399 | 1531 |
| N50 contig length | 26,244,591 | 46,208,332 |
| contig L50 | 32 | 20 |
| Number of scaffolds | 29,907 | 1362 |
| N50 scaffold length (bp) | 87,277,232 | 102,383,509 |
| Scaffold L50 | 13 | 11 |

**Table S4. Repeat content of Saanen_v1 as compared with ARS1.** The unplaced scaffolds from each assembly were not included for comparison.

|  |  | **Saanen_v1** | | | **ARS1** | | |
| --- | --- | --- | --- | --- | --- | --- | --- |
| **Repeat class** | **Repeat subtype** | **Number of elements** | **Length, bp** | **% of sequence** | **Number of elements** | **Length, bp** | **% of sequence** |
| SINEs |  | 2,036,635 | 297,324,302 | 11.31 | 2,009,190 | 293,558,140 | 11.37 |
|  | MIRs | 389,833 | 55,800,399 | 2.12 | 385,151 | 55,180,243 | 2.14 |
| LINEs |  | 1,323,275 | 739,315,136 | 28.13 | 1,302,967 | 725,784,532 | 28.11 |
|  | LINE1 | 587,710 | 333,688,885 | 12.70 | 577,147 | 326,644,751 | 12.65 |
|  | LINE2 | 247,823 | 61,999,428 | 2.36 | 245,311 | 61,316,365 | 2.37 |
|  | L3/CR1 | 33,000 | 6,800,923 | 0.26 | 32,697 | 6,753,059 | 0.26 |
|  | RTE | 453,694 | 336,664,233 | 12.81 | 446,782 | 330,913,627 | 12.82 |
| LTR elements |  | 409,627 | 125,832,666 | 4.79 | 402,109 | 123,148,053 | 4.77 |
|  | ERVL | 75,906 | 29,245,065 | 1.11 | 74,941 | 28,842,261 | 1.12 |
|  | ERVL-MaLRs | 122,524 | 39,549,104 | 1.50 | 120,865 | 39,082,093 | 1.51 |
|  | ERV_classI | 83,345 | 35,728,161 | 1.36 | 81,076 | 34,642,101 | 1.34 |
|  | ERV_classII | 110,466 | 17,232,768 | 0.66 | 107,949 | 16,527,062 | 0.64 |
| DNA elements |  | 290,040 | 57,245,301 | 2.18 | 286,920 | 56,615,684 | 2.19 |
|  | hAT-Charlie | 163,769 | 30,376,532 | 1.16 | 161,951 | 30,065,234 | 1.16 |
|  | TcMar-Tigger | 44,506 | 11,789,218 | 0.45 | 44,188 | 11,650,992 | 0.45 |
| Unclassified |  | 5284 | 887,076 | 0.03 | 5245 | 883,294 | 0.03 |
| Total interspersed repeats |  |  | 1,220,604,481 | 46.44 |  | 1,199,989,703 | 46.47 |
| Satellites |  | 2576 | 4,065,142 | 0.15 | 1761 | 1,668,877 | 0.06 |
| Simple repeats |  | 515,948 | 22,281,523 | 0.85 | 508,989 | 21,723,858 | 0.84 |
| Low complexity |  | 81,573 | 4,034,348 | 0.15 | 80,716 | 3,987,876 | 0.15 |

**Table S5. Telomere signals identified in each assembly**

| CHR | Saanen_v1 (bp) | ARS1 (bp) | Sheep (bp) | Cattle (bp) |
| --- | --- | --- | --- | --- |
| 1 | 9940 | 0 | 12,347 | 0 |
| 2 | 0 | 0 | 0 | 0 |
| 3 | 0 | 0 | 0 | 0 |
| 4 | 0 | 0 | 0 | 0 |
| 5 | 0 | 0 | 0 | 7233 |
| 6 | 13,717 | 0 | 4444 | 0 |
| 7 | 11,502 | 5318 | 10,510 | 0 |
| 8 | 13,891 | 0 | 0 | 0 |
| 9 | 7926 | 5195 | 0 | 0 |
| 10 | 14,353 | 24,448 | 0 | 0 |
| 11 | 16,286 | 0 | 9840 | 0 |
| 12 | 18,575 | 0 | 0 | 0 |
| 13 | 6611 | 0 | 8496 | 4929 |
| 14 | 11,734 | 0 | 0 | 0 |
| 15 | 11,585 | 0 | 0 | 0 |
| 16 | 2424 | 0 | 0 | 8032 |
| 17 | 10,066 | 0 | 0 | 0 |
| 18 | 4767 | 4698 | 0 | 2220 |
| 19 | 12,832 | 2180 | 0 | 0 |
| 20 | 14,488 | 2421 | 13,956 | 0 |
| 21 | 0 | 0 | 0 | 0 |
| 22 | 5029 | 0 | 0 | 0 |
| 23 | 18,806 | 2053 | 0 | 0 |
| 24 | 8188 | 0 | 0 | 0 |
| 25 | 14,990 | 0 | 0 | 0 |
| 26 | 16,511 | 0 | 0 | 0 |
| 27 | 0 | 0 | NA | 0 |
| 28 | 0 | 0 | NA | 0 |
| 29 | 18,141 | 0 | NA | 0 |
| Y | 5497 | 0 | NA | 0 |
| X | 5310 | 0 | 0 | 2723 |
| Sum | 24/31 | 7/31 | 6/27 | 5/31 |

**Table S6. Centromere signals identified in each assembly**

| CHR | Saanen_v1 (bp) | ARS1 (bp) | Sheep (bp) | Cattle (bp) |
| --- | --- | --- | --- | --- |
| 1 | 78,185 | 31,956 | 33,099 | 18,4373 |
| 2 | 102,347 | 0 | 99,441 | 197,971 |
| 3 | 0 | 0 | 57,239 | 0 |
| 4 | 185,690 | 0 | 18,525 | 298,605 |
| 5 | 60,574 | 56,168 | 6238 | 43,063 |
| 6 | 33,216 | 39,404 | 53,596 | 0 |
| 7 | 0 | 0 | 43,548 | 6395 |
| 8 | 56,404 | 14,757 | 35,423 | 16,922 |
| 9 | 39,965 | 18,947 | 8152 | 6263 |
| 10 | 86,773 | 54,330 | 5365 | 53,085 |
| 11 | 443,333 | 0 | 0 | 108,333 |
| 12 | 69,389 | 21,458 | 0 | 107,124 |
| 13 | 39,142 | 48,363 | 70,494 | 85,158 |
| 14 | 39,242 | 0 | 26,790 | 0 |
| 15 | 0 | 0 | 111,556 | 173,769 |
| 16 | 46,189 | 0 | 0 | 134,684 |
| 17 | 67,534 | 63,110 | 146,604 | 23,999 |
| 18 | 9044 | 0 | 57,964 | 57,679 |
| 19 | 14,311 | 99,969 | 66,805 | 53,070 |
| 20 | 113,312 | 47,477 | 76,415 | 0 |
| 21 | 41,094 | 12,703 | 40,516 | 9782 |
| 22 | 132,090 | 35,408 | 23,775 | 0 |
| 23 | 44,574 | 46,395 | 19,663 | 0 |
| 24 | 157,058 | 10,425 | 0 | 0 |
| 25 | 26,810 | 9854 | 0 | 0 |
| 26 | 37,208 | 46,491 | 31,598 | 15,126 |
| 27 | 186,965 | 35,552 | NA | 59,271 |
| 28 | 5037 | 5052 | NA | 32,553 |
| 29 | 22,004 | 14,946 | NA | 32,107 |
| Y | 0 | 0 | 0 | 0 |
| X | 86,180 | 5961 | 14,290 | 0 |
| Sum | 2,223,670 | 718,726 | 1,047,096 | 1,699,332 |

Note: The assemblies of goat (ARS1, GCA_001704415.1), sheep (Oar_rambouillet_v1.0, GCA_002742125.1) and cattle (ARS-UCD1.2, GCA_002263795.2) were used for comparison.

**Table S7. Structural inconsistencies when comparing Saanen_v1 and ARS1 assemblies**

| **Catergory** | **Saanen_v1** | **ARS1** | **Description** |
| --- | --- | --- | --- |
| COMPR_PE | 112,540 | 174,013 | Areas with Low CE statistics |
| STRECH_PE | 1,426,906 | 1,572,772 | Areas with high CE statistics |
| HIGH_COV_PE | 6846 | 6077 | High read coverage areas (all aligned reads) |
| HIGH_NORM_COV_PE | 6382 | 5487 | High read coverage areas (only properly aligned pairs) |
| HIGH_OUTIE_PE | 176 | 127 | Regions with high numbers of misoriented or distant pairs |
| HIGH_SINGLE_PE | 34 | 75 | Regions with high numbers of unmapped pairs |
| HIGH_SPAN_PE | 3141 | 5224 | Regions with high number of pairs that map to different scaffolds |
| LOW_COV_PE | 140,185 | 206,855 | Low read coverage areas (all aligned reads) |
| LOW_NORM_COV_PE | 140,278 | 206,071 | Low paired-end coverage areas (only properly aligned pairs) |

**Table S8. Mapping ratio of whole genome sequencing data**

| **Sample_ID** | **Saanen_v1** | | **ARS1** | | **Total reads** | **SRA** |
| --- | --- | --- | --- | --- | --- | --- |
|  | **Mapping ratio** | **Mapped reads** | **Mapping ratio** | **Mapped reads** |  |  |
| P1 | 97.06% | 391,942,242 | 97.10% | 392,098,840 | 403,809,398 | SRR5803174 |
| ERR313197 | 98.59% | 358,670,253 | 98.55% | 358,518,915 | 363,792,304 | ERR313197 |
| ERR313206 | 98.99% | 353,596,337 | 98.95% | 353,437,488 | 357,186,094 | ERR313206 |
| ERR313211 | 98.78% | 381,733,921 | 98.74% | 381,568,891 | 386,444,624 | ERR313211 |
| ERR313213 | 98.74% | 384,073,161 | 98.70% | 383,906,960 | 388,973,310 | ERR313213 |

**Table S9. Mapping ratio of RNA-seq data**

| **Sample_ID** | **Saanen_v1** | **ARS1** | **Total_reads** | **Tissue** | **SRA** |
| --- | --- | --- | --- | --- | --- |
| muscle1 | 97.08% | 96.88% | 50,303,756 | muscle | SRR1822383 |
| muscle2 | 97.22% | 96.99% | 46,619,080 | muscle | SRR1822384 |
| muscle3 | 97.41% | 97.21% | 48,019,522 | muscle | SRR1822385 |
| lung1 | 96.67% | 96.32% | 41,816,060 | lung | SRR1822386 |
| lung2 | 96.19% | 96.04% | 50,402,296 | lung | SRR1822387 |
| lung3 | 97.00% | 96.85% | 50,365,054 | lung | SRR1822388 |
| heart1 | 97.36% | 96.92% | 46,179,396 | heart | SRR1822389 |
| heart2 | 96.89% | 97.09% | 48,017,740 | heart | SRR1822390 |
| heart3 | 97.24% | 97.00% | 48,286,532 | heart | SRR1822391 |

**Table S10. Mapping ratio of Iso-seq data**

| **Sample_ID** | **Saanen_v1** | | **ARS1** | | **Total reads** | **SRA** |
| --- | --- | --- | --- | --- | --- | --- |
|  | **Mapping ratio** | **Mapped reads** | **Mapping ratio** | **Mapped reads** |  |  |
| Isoseq-1 | 92.87% | 21,695 | 92.33% | 21,568 | 23,360 | SRR8618141 |
| Isoseq-2 | 93.95% | 19,137 | 93.46% | 19,038 | 20,370 | Unpublished |
| Isoseq-3 | 93.01% | 20,707 | 92.38% | 20,567 | 22,264 | Unpublished |
| Isoseq-4 | 96.20% | 28,343 | 94.73% | 27,911 | 29,464 | SRR11410765 |

**Table S11. Summary of structural variations in Saanen_v1 as compared with ARS1**

|  | 50-500 bp | | 500 bp-10000 bp | | Total | |
| --- | --- | --- | --- | --- | --- | --- |
|  | Count | Total bp | Count | Total bp | Count | Total bp |
| Insertion | 3727 | 610,784 | 1454 | 2,605,935 | 5181 | 3,216,719 |
| Deletion | 4521 | 639,374 | 1366 | 2,555,980 | 5887 | 3,195,354 |
| Repeat expansion | 555 | 125,187 | 471 | 1,102,349 | 1026 | 1,227,536 |
| Repeat contraction | 562 | 127,168 | 666 | 1,599,384 | 1228 | 1,726,552 |
| Tandem expansion | 1252 | 230,321 | 417 | 844,132 | 1669 | 1,074,453 |
| Tandem contraction | 1292 | 229,586 | 431 | 1,062,975 | 1723 | 1,292,561 |

**Table S13. Blast alignment of previously reported goat Y chromosome amplicons to the Y chromosome assembly included in Saanen_v1**

| **Gene** | **GenBank ID** | **Length (bp)** | **Identity** | **Coverage** | **Reference** |
| --- | --- | --- | --- | --- | --- |
| AMELY | MF448228 | 346 | 100 | 100 | Vidal et al., 2017, Scientific Reports 7(1):16161 |
| DDX3Y | MF448230 | 166 | 98.8 | 100 | Vidal et al., 2017, Scientific Reports 7(1):16161 |
| DDX3Y | MF448232 | 520 | 100 | 100 | Vidal et al., 2017, Scientific Reports 7(1):16161 |
| SRY | D82963.1 | 586 | 99.3 | 100 | Waki et al., 2015, Animal Genetics, 46(3):337-339 |
| SRY | LC416985 | 478 | 99.8 | 100 | Tabata et al., 2018, Animal Science Journal 90(3), 317-322 |
| UTY | MF448235 | 217 | 100 | 100 | Vidal et al., 2017, Scientific Reports 7(1):16161 |
| ZFY | AY082500 | 927 | 98.7 | 100 | Pidancier et al., 2006, Molecular Phylogenetics and Evolution 40(3), 739-749 |
| ZFY | MF448234 | 629 | 99.8 | 100 | Vidal et al., 2017, Scientific Reports 7(1):16161 |

**Table S15. Information of large gap regions (>1 Mb) between two adjacent SNP probes**

| Chr | SNP | Position (bp) | SNP | Position (bp) | Distance (bp) |
| --- | --- | --- | --- | --- | --- |
| 4 | snp46420-scaffold641-1426922 | 3,448,376 | snp15036-scaffold1609-17621 | 4,121,998 | 673,622 |
| 5 | snp54201-scaffold827-2159757 | 90,649,066 | snp47384-scaffold666-1627339 | 95,558,653 | 4,909,587 |
| 8 | snp32818-scaffold38-2453898 | 93,809,629 | snp32817-scaffold38-2422121 | 95,311,218 | 1,501,589 |
| 9 | snp33825-scaffold3987-12018 | 74,515,422 | snp27534-scaffold2932-48964 | 75,290,173 | 774,751 |
| 12 | snp30121-scaffold3305-540 | 72,485,226 | snp21490-scaffold21-42481 | 73,106,612 | 621,386 |
| 13 | snp19347-scaffold1952-562068 | 80,049,795 | snp22420-scaffold2213-270881 | 81,133,791 | 1,083,996 |
| 22 | snp18463-scaffold1869-624798 | 55,417,257 | snp12244-scaffold1458-16655 | 57,652,022 | 2,234,765 |
